# Low-Level Light Therapy for Dry Eye Disease in Thyroid Eye Disease: A Randomized, Double-Masked, Sham-Controlled Trial

**DOI:** 10.3390/ijms27219802

**Published:** 2026-10-31

**Authors:** Mihaela-Madalina Timofte-Zorila, Alin Constantin Pînzariu, Mariana Pavel-Tanasa, Giuseppe Giannaccare, Sinziana Istrate, Nicoleta Vlas, Diana Alecu, Tudor-Stefan Rotaru, Radu Tanasa, Filippo Lixi, Mario Troisi, Cristina Preda, Daniel Constantin Branisteanu

**Affiliations:** 1 Grigore T. Popa University of Medicine and Pharmacy Iasi, 700115 Iasi, Romania; mihaela-madalina.timofte-zorila@d.umfiasi.ro (M.-M.T.-Z.); vlas.nicoleta@email.umfiasi.ro (N.V.); alecu.diana@email.umfiasi.ro (D.A.); tudor.rotaru@umfiasi.ro (T.-S.R.); cristina.preda@umfiasi.ro (C.P.); daniel.branisteanu@umfiasi.ro (D.C.B.); 2 Ophthalmology Clinic, Cai Ferate Clinical Hospital, 700506 Iasi, Romania; 3 Laboratory of Immunology, St. Spiridon County Clinical Hospital, 700111 Iasi, Romania; 4 Advanced Research and Development Center for Experimental Medicine “Prof. Ostin C. Mungiu”—CEMEX, Grigore T. Popa University of Medicine and Pharmacy Iasi, 700259 Iasi, Romania; 5 Eye Clinic, Department of Surgical Sciences, University of Cagliari, 09124 Cagliari, Italy; giuseppe.giannaccare@unica.it (G.G.);; 6 Carol Davila University of Medicine and Pharmacy, 050474 Bucharest, Romania; sanzinici@yahoo.com; 7 Optissima, Barbu Văcărescu 102, 020283 Bucharest, Romania; 8 Faculty of Physics, Alexandru Ioan Cuza University, 700506 Iasi, Romania; radu.tanasa@uaic.ro; 9 Department of Medical Sciences and Public Health, University of Cagliari, 09124 Cagliari, Italy; 10 Eye Clinic, Department of Neurosciences, Reproductive and Odontostomatological Sciences, Federico II University, 80131 Naples, Italy; mario.troisi@sangiovannieruggi.it; 11 Ophthalmologic Unit, Salerno University Hospital, 84131 Salerno, Italy

**Keywords:** low-level light therapy, photobiomodulation, dry eye disease, thyroid eye disease, Graves’ ophthalmopathy, tear osmolarity, ocular surface disease index

## Abstract

Dry eye disease (DED) affects the majority of patients with thyroid eye disease (TED) and frequently persists despite maximal conservative therapy. Low-level light therapy (LLLT), or photobiomodulation, is a non-invasive treatment with anti-inflammatory and pro-reparative properties whose value in autoimmune, chronically driven ocular surface disease is unknown. In this prospective, randomized, double-masked, sham-controlled trial, eyes of patients with TED-associated DED that remained symptomatic despite intensive preservative-free lubrication, bedtime ophthalmic ointment and daily periocular hygiene were allocated 2:1 to periocular LLLT (630 nm LED mask, 15 min per session, applied over closed eyelids) or to sham treatment (identical procedure with light emission reduced to <30% of therapeutic output). Background ocular surface therapy was maintained unchanged in both arms. Assessments were performed at baseline (T0), weekly during treatment (T1–T3) and at one month (T4) and two months (T5) after the last session. The primary outcome was the Ocular Surface Disease Index (OSDI); secondary outcomes were non-invasive break-up time (NIBUT), fluorescein break-up time (FBUT), Schirmer I test, tear osmolarity, upper- and lower-eyelid meibography, corneal fluorescein staining and lipid-layer grade. The primary OSDI assessment was the change from baseline to T3, the end-of-treatment visit. Additional follow-up assessments were performed at T4 and T5. Forty-six eyes of 46 patients were analyzed (30 LLLT, 16 sham); baseline disease was active in 14/30 (46.7%) and 6/16 (37.5%) eyes, respectively. At T3, adjusted OSDI was lower with LLLT than with sham (between-group difference −7.49 points; 95% CI −11.66 to −3.32; *p* < 0.001). The difference persisted at T4 and T5 (−11.97 and −16.86 points, respectively; both Holm-adjusted *p* < 0.001). Tear osmolarity was lower with LLLT at T4 (−10.91 mOsm/L; 95% CI −18.96 to −2.87; *p* = 0.009) and T5 (−13.81 mOsm/L; 95% CI −22.55 to −5.06; *p* = 0.004). No multiplicity-adjusted between-group differences were demonstrated for NIBUT, FBUT, Schirmer I test or meibography. The adjusted probability of corneal staining was numerically lower with LLLT (T4: 15.7% vs. 34.7%; risk difference −19.0 percentage points; *p* = 0.100). In exploratory analyses, the treatment-by-visit-by-activity interaction was significant (*p* = 0.0159), with OSDI differences of −21.45 and −25.90 points in active disease versus −5.50 and −10.09 points in inactive disease at T4 and T5. CAS and OSDI were positively correlated at baseline (Spearman ρ = 0.547; Holm-adjusted *p* < 0.001) and at T5 (Spearman ρ = 0.428; Holm-adjusted *p* = 0.003). No treatment-related adverse events occurred. In conclusion, in TED-associated DED refractory to maximal conservative therapy, LLLT produced a significant and progressively increasing reduction in symptom burden together with an objective reduction in tear hyperosmolarity. Effects on break-up time, aqueous secretion, gland morphology, and lipid layer grade were not demonstrable within two months. The pattern and pace of response differ from those observed with the same technology after cataract surgery and are consistent with a chronic, systemically driven disease in which the ocular surface insult is continuously renewed.

## 1. Introduction

Thyroid eye disease (TED), also referred to as Graves’ orbitopathy, is an autoimmune condition that develops in almost half of patients with Graves’ disease [1]. The orbital reaction is a direct consequence of cross-reactivity against the thyroid-stimulating hormone receptor (TSHR), an antigen expressed both in thyroid tissue and in orbital fibroblasts [2,3]. The resulting inflammatory process leads to glycosaminoglycan deposition, adipogenesis and collagen accumulation, with enlargement and fibrosis of the orbital fat and extraocular muscles [1,4]. Disease activity is graded using the Clinical Activity Score (CAS), with CAS ≥ 3/7 defining active disease, whereas CAS < 3 indicates inactive disease; severity is graded separately according to European Group on Graves’ Orbitopathy (EUGOGO) criteria [4,5]. Proptosis, eyelid retraction and lagophthalmos are the typical features, and even subclinical ocular alterations may precede overt orbitopathy [5,6].

These mechanical and inflammatory changes converge on the ocular surface and make dry eye disease (DED) one of the most frequent complications of TED, reported in 60–80% of cases [7,8]. The distinction between active and inactive disease is clinically relevant, because activity correlates not only with inflammatory burden but also with the severity of ocular surface involvement. Ocular discomfort, burning, foreign body sensation, photophobia and light discomfort all contribute to a substantial reduction in quality of life in this population [8,9]. Although lubricants, topical ciclosporin and corticosteroids are available, a considerable proportion of patients remain symptomatic despite maximal conservative therapy, and the management of ocular surface disease in the presence of TED remains a challenge [10,11,12].

Low-level light therapy (LLLT), also known as photobiomodulation, uses low-irradiance light to promote tissue regeneration, modulate inflammation and relieve pain. At the cellular level it acts principally through cytochrome c oxidase in the mitochondrial respiratory chain, increasing oxidative metabolism and adenosine triphosphate availability and activating downstream signaling pathways involved in repair and in the resolution of inflammation [13,14]. The technique is non-invasive, well tolerated and has been applied across dermatology, physical medicine and, increasingly, ophthalmology [15].

Most of the ophthalmic evidence for LLLT, however, has been generated in evaporative DED and in the perioperative setting of cataract surgery, where the ocular surface insult is acute, iatrogenic and self-limiting. Whether the same technology retains its efficacy when the ocular surface is the downstream target of a chronic, systemically driven autoimmune process has not been established. The present randomized, double-masked, sham-controlled trial was therefore designed to evaluate the effect of LLLT on ocular surface parameters and ocular discomfort symptoms in patients with TED-associated DED that had proved refractory to intensive conventional treatment.

## 2. Results

### 2.1. Baseline Characteristics and Data Availability

Sixty patients were screened for eligibility; nine did not meet the inclusion criteria or declined to participate, and 51 were randomized. After randomization, two patients in the treatment group discontinued the intervention for personal reasons related to the frequency of study visits, and one patient was lost to follow-up. In the sham group, two patients were lost to follow-up. None of these five patients (three LLLT, two sham) had evaluable post-baseline data, and all five were therefore excluded from the analysis population.

**Figure 1:**
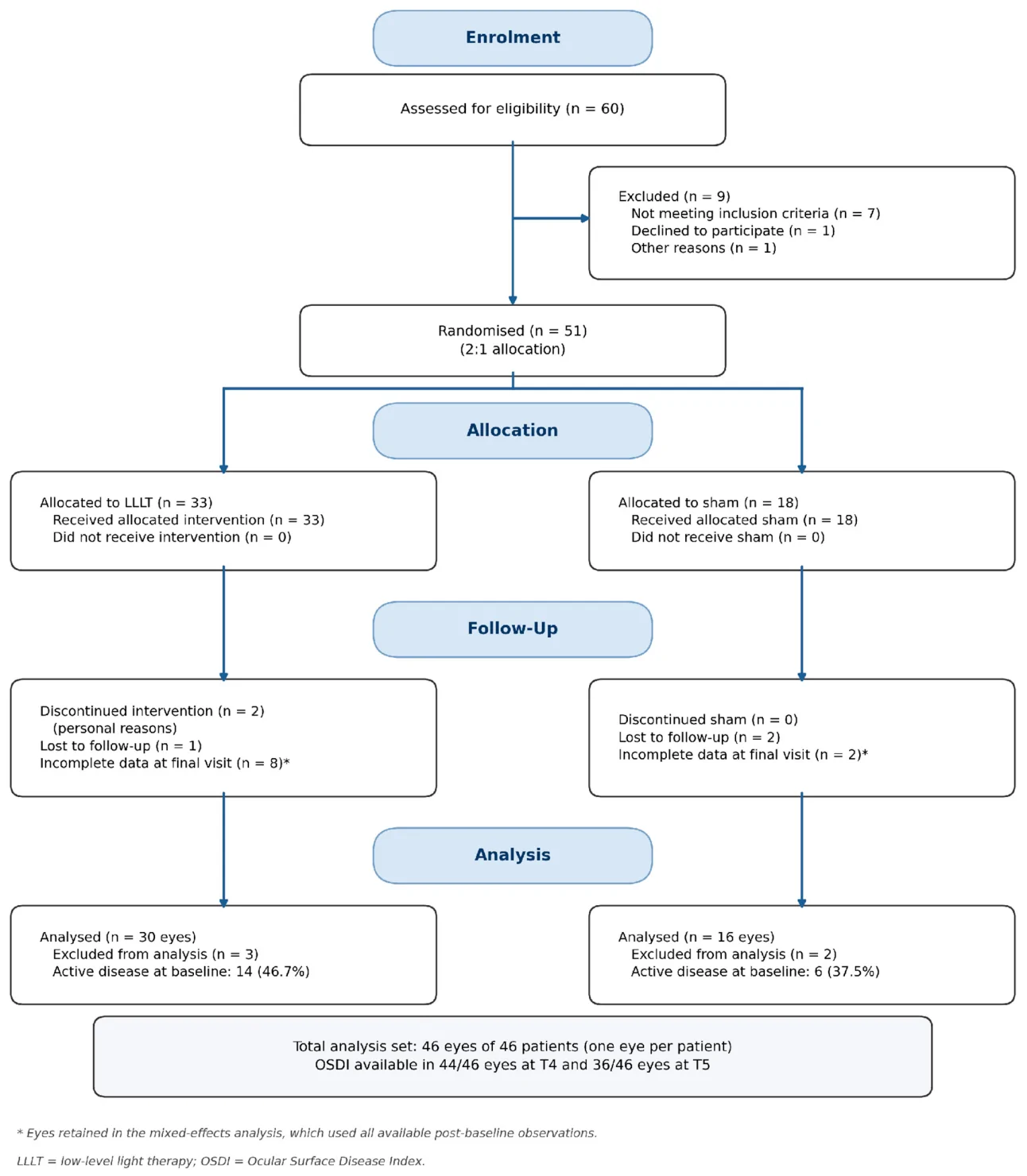
CONSORT flow diagram. Progress of participants through the phases of the trial, from screening to the analysis set of 46 eyes of 46 patients (30 low-level light therapy, 16 sham).

The analysis set comprised 46 eyes of 46 patients: 30 in the LLLT group and 16 in the sham group. Participant flow is summarized in Figure 1. All 46 participants completed OSDI at every visit from T0 to T3; at T4 and T5, 44 and 36 questionnaires, respectively, met the completeness criteria and were analyzed (Section 4.6).

Baseline disease was active in 6/16 (37.5%) sham eyes and 14/30 (46.7%) LLLT eyes. Baseline characteristics are shown in Table 1. Age and sex distribution were comparable, whereas baseline imbalance was most pronounced for the quality-of-life score (SMD 0.82; *p* = 0.030), primary gaze intraocular pressure (SMD 1.00; *p* = 0.003) and exophthalmometry (SMD 0.66; *p* = 0.041). All subsequent longitudinal treatment comparisons were therefore interpreted from baseline-adjusted models. Longitudinal treatment comparisons were adjusted for the baseline value of the corresponding outcome. This adjustment did not account for all baseline differences between treatment groups.

### 2.2. Primary Outcome: Ocular Surface Disease Index

At the end of treatment (T3), the mean OSDI score was 19.47 ± 9.05 in the LLLT group and 24.25 ± 11.11 in the sham group. The mean reduction in OSDI from baseline to T3 was 13.67 points in the LLLT group, compared with 3.69 points in the sham group. In the baseline-adjusted longitudinal mixed effects model, the estimated between-group difference in OSDI at T3 (LLLT minus sham) was −7.49 points (95% CI, −11.66 to −3.32; *p* < 0.001), favoring LLLT. Although the unadjusted between-group difference in change exceeded the 8-point threshold used for sample size planning, the adjusted point estimate was slightly below this threshold.

**Figure 2:**
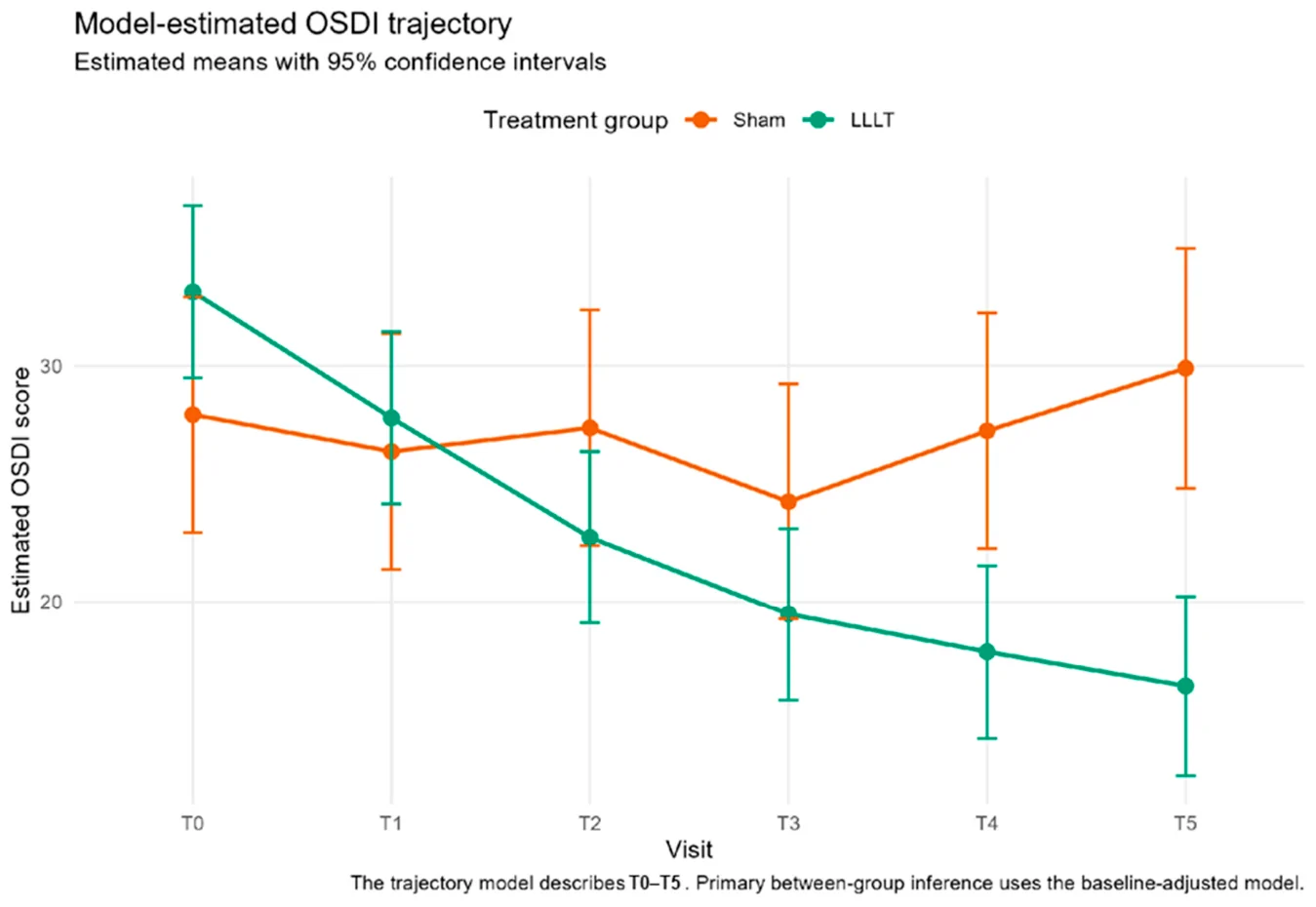
Ocular Surface Disease Index (OSDI) trajectory across time points. Observed baseline means and model-estimated post-baseline marginal means are shown; dots represent means and bars 95% confidence intervals. The originally specified primary between-group comparison was the change in OSDI from baseline to T3. The contrasts at T4 and T5 represent additional follow-up assessments.

At the additional follow-up assessments, the treatment-by-visit interaction for OSDI was significant (*p* < 0.001). Adjusted OSDI was lower with LLLT than with sham at both additional follow-up assessments: the mean difference was −11.97 points at T4 (95% CI −16.44 to −7.50; Holm-adjusted *p* < 0.001) and −16.86 points at T5 (95% CI −21.51 to −12.21; Holm-adjusted *p* < 0.001). Within the LLLT group, adjusted changes from baseline were −15.27 points at T4 (95% CI −18.10 to −12.44) and −16.72 points at T5 (95% CI −19.77 to −13.67), whereas the sham group showed relatively constant scores throughout follow-up (Figure 2). Both endpoint differences exceed by a wide margin the 8-point difference considered clinically meaningful for OSDI.

The T4 and T5 estimates were robust to the analytical approach (Supplementary Table S1 and Supplementary Figure S1). Complete-case estimates were −12.63 points at T4 (95% CI −17.46 to −7.81) and −16.65 points at T5 (95% CI −21.47 to −11.82), and separate ANCOVA estimates were −11.76 points at T4 (95% CI −16.90 to −6.63) and −16.65 points at T5 (95% CI −21.81 to −11.49). Residual diagnostics were acceptable, with no standardized residual exceeding ±3 (Shapiro–Wilk *p* = 0.125). The close agreement between the primary mixed model, the complete-case model and the endpoint-specific ANCOVA analyses supports the reliability of these estimates. Additional adjustment for age, baseline CAS, and baseline quality-of-life score yield-ed LLLT-minus-sham differences of −12.4 OSDI points at T4 (95% CI −16.8 to −8.0) and −17.3 points at T5 (95% CI −21.9 to −12.6; both Holm-adjusted *p* < 0.001). These estimates were similar to those from the original model.

In the separate sensitivity analysis adjusting for baseline quality of life, primary gaze intraocular pressure, and ophthalmometry, the estimated differences were −12.6 points at T4 (95% CI −17.7 to −7.6) and −17.5 points at T5 (95% CI −22.7 to −12.4; both Holm-adjusted *p* < 0.001). These estimates were similar to those from the original model, although the confidence intervals were wider.

### 2.3. Secondary Outcomes

**Figure 3:**
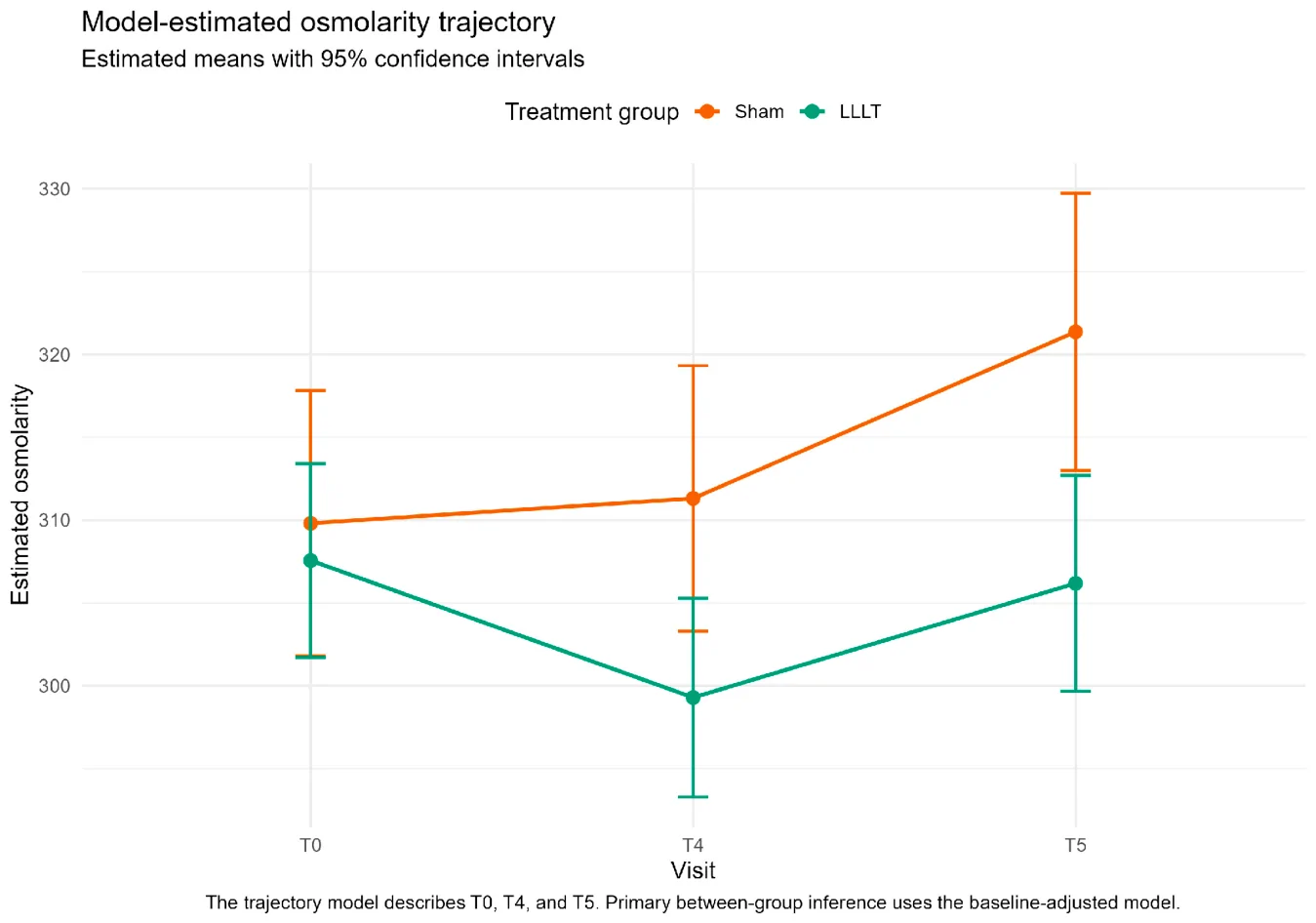
Tear osmolarity trajectory across time points. Observed baseline means and model-estimated post-baseline marginal means; dots represent means and bars 95% confidence intervals. Between-group estimates were baseline-adjusted.

Baseline-adjusted between-group differences for all secondary outcomes at T4 and T5 are presented in Table 2. LLLT was associated with lower tear osmolarity at both endpoints, −10.91 mOsm/L at T4 (95% CI −18.96 to −2.87; Holm-adjusted *p* = 0.009) and −13.81 mOsm/L at T5 (95% CI −22.55 to −5.06; Holm-adjusted *p* = 0.004), reflecting a shift from hyperosmolar values towards the physiological range in treated eyes (Figure 3).

No multiplicity-adjusted between-group differences were detected for NIBUT, FBUT, the Schirmer test or upper- and lower-eyelid meibography (Supplementary Figures S2–S5). For these outcomes, original-scale, log-scale and influence analyses were compared where warranted, and sensitivity analyses generally yielded conclusions consistent with the primary analyses. The nominal FBUT difference at T4 (+1.16 s; 95% CI 0.11 to 2.21) and the lower-eyelid meibography difference at T5 (−4.39; 95% CI −8.46 to −0.33) did not remain significant after Holm adjustment and were not robust across sensitivity analyses; both should be regarded as hypothesis-generating and merit evaluation in larger studies.

#### 2.3.1. Lipid Layer Grade

Tear film lipid layer grade (LLG) was assessed using the DEA 520 automated ocular surface analyzer (Shanghai MediWorks Precision Instruments Co., Ltd., Shanghai, China), evaluated by interferometric pattern analysis, and graded according to the standard lipid layer templates provided by the device (Grade 1: <30 nm; Grade 2: 30–60 nm; Grade 3: 60–80 nm; Grade 4: >80 nm). At baseline (T0), the Sham and LLLT groups showed comparable distributions of lipid layer grades, with Grade 2 and Grade 3 predominating in both groups. No statistically significant differences between the two groups were observed at T0 or at any subsequent assessment point (T1–T5; all *p* > 0.05). Although a greater proportion of eyes in the LLLT group shifted toward higher lipid layer grades at T4 and T5, including the emergence of Grade 4, these differences did not reach statistical significance.

**Figure 4:**
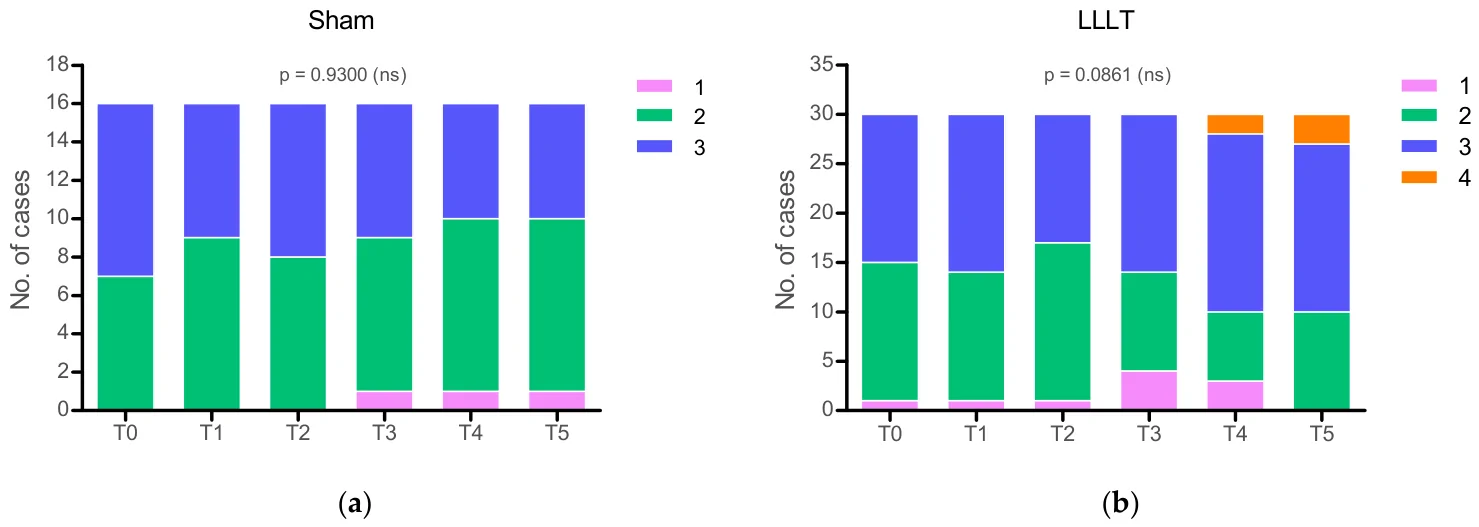
Observed distribution of lipid layer grade from T0 to T5, by treatment group: (**a**) Sham and (**b**) LLLT (ns—not significant; chi-squared test).

Within-group analysis also showed no statistically significant changes in the distribution of lipid layer grades over time in either the sham group (*p* = 0.930, Figure 4a) or the LLLT group (*p* = 0.086, Figure 4b). Thus, while the LLLT group demonstrated a descriptive trend toward higher lipid layer grades during the later follow-up assessments, the observed changes were not statistically significant.

#### 2.3.2. Corneal Fluorescein Staining

**Figure 5:**
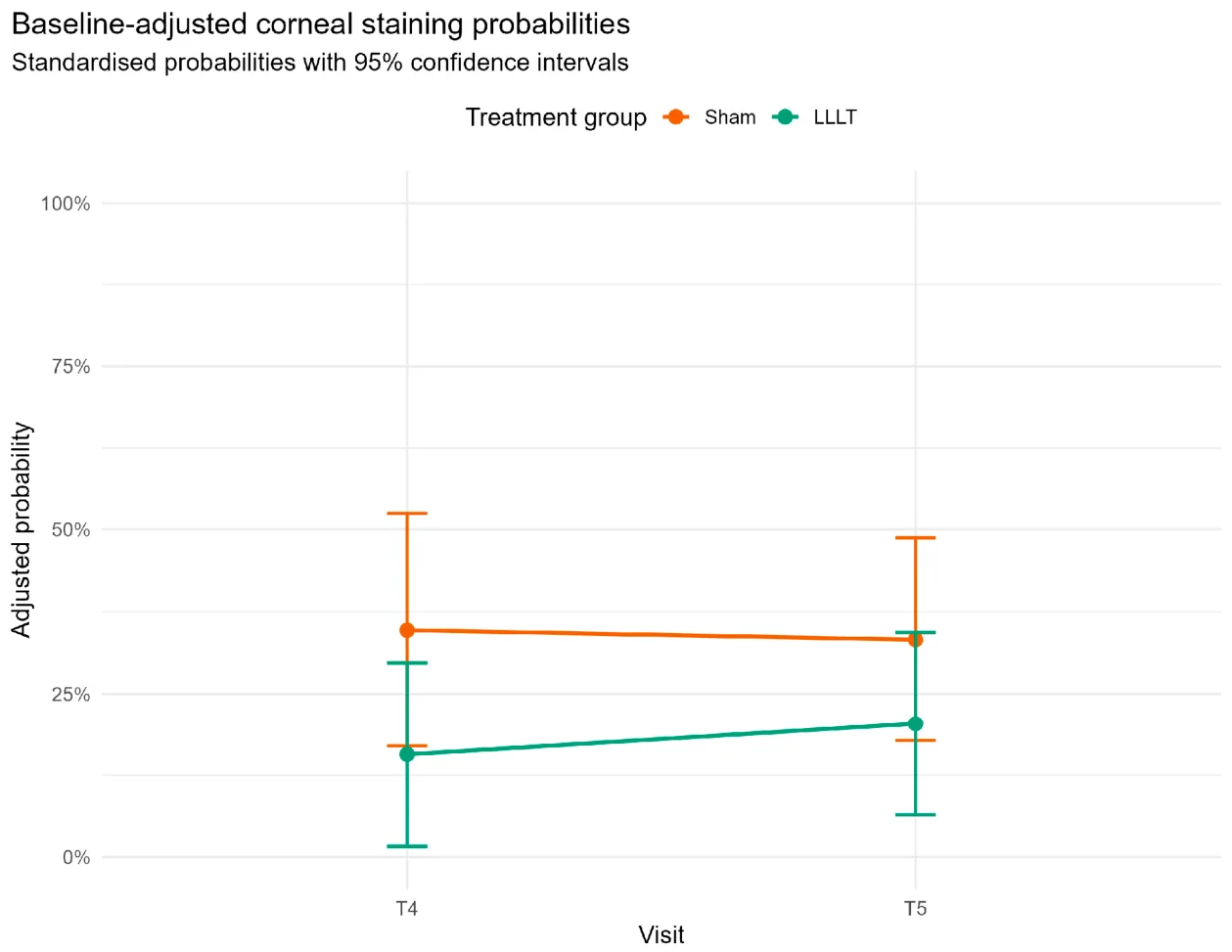
Baseline-adjusted probabilities of corneal fluorescein staining at T4 and T5. Dots represent the standardized probability and bars 95% confidence intervals.

Separate mean bias-reduced logistic regression models, adjusted for baseline staining presence, were used at T4 and T5. At T4, the standardized probability of corneal staining was 34.7% (95% CI 16.9–52.4) with sham and 15.7% (95% CI 1.6–29.7) with LLLT, giving a risk difference of −19.0 percentage points (95% CI −41.6 to 3.6; *p* = 0.100) and an OR of 0.22 (95% CI 0.03–1.59; *p* = 0.133). At T5, the corresponding probabilities were 33.2% (95% CI 17.8–48.7) and 20.4% (95% CI 6.4–34.3), with a risk difference of −12.9 percentage points (95% CI −33.7 to 8.0; *p* = 0.226) and an OR of 0.28 (95% CI 0.03–2.53; *p* = 0.256) (Figure 5). The direction of effect consistently favored LLLT at both endpoints, but the estimates were imprecise and no statistically significant difference was demonstrated.

### 2.4. Exploratory Effect Modification by Baseline Disease Activity

**Figure 6:**
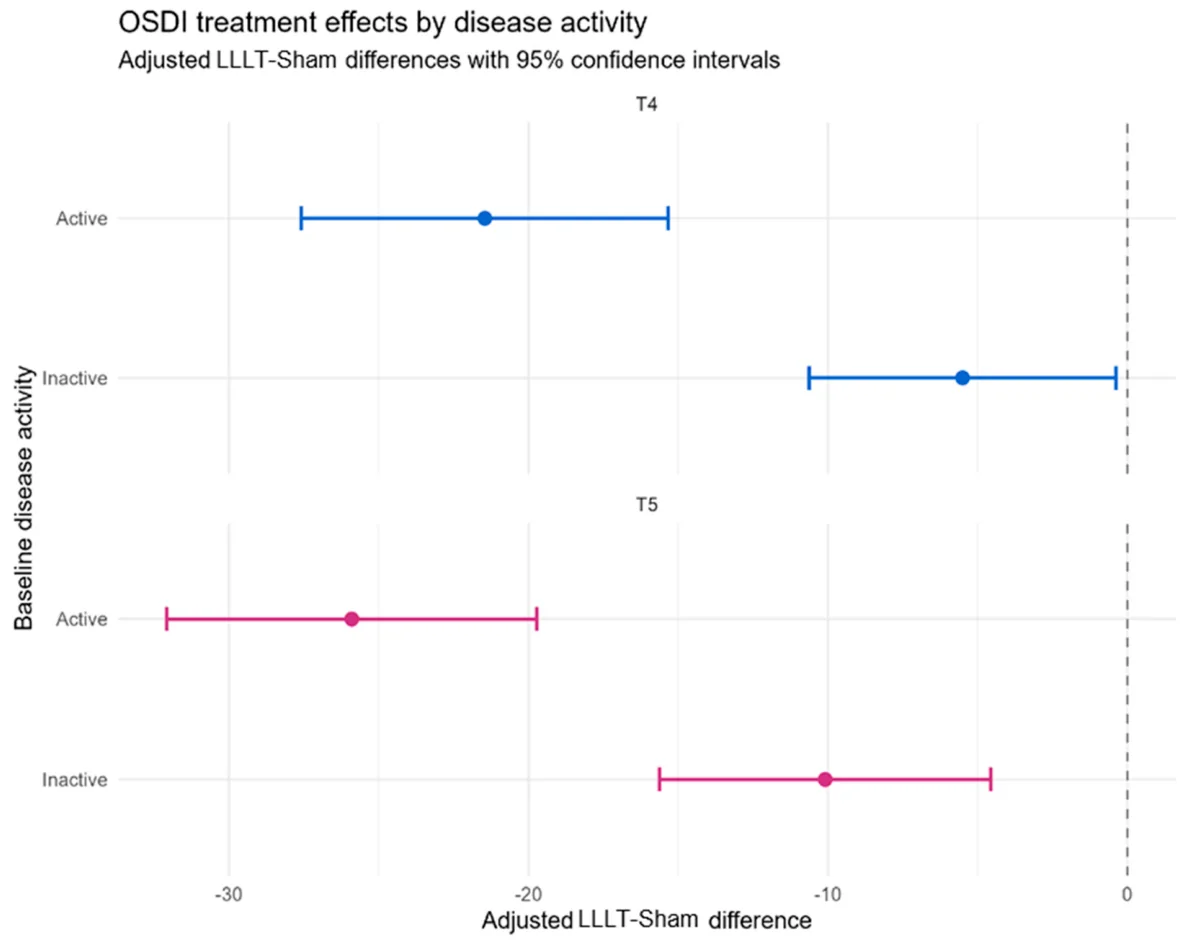
Exploratory treatment effects on OSDI stratified by baseline disease activity. Dots represent the adjusted LLLT-minus-sham difference and bars 95% confidence intervals. The active sham subgroup comprised only six eyes.

In inactive disease, adjusted LLLT-minus-sham OSDI differences were −5.50 points at T4 (95% CI −10.62 to −0.38) and −10.09 points at T5 (95% CI −15.62 to −4.56). In active disease, the corresponding differences were −21.45 points (95% CI −27.58 to −15.33) and −25.90 points (95% CI −32.08 to −19.71). The active-minus-inactive differences in treatment effect were −15.95 points at T4 (95% CI −23.85 to −8.06) and −15.81 points at T5 (95% CI −23.99 to −7.62) (Figure 6). These analyses were exploratory and must be interpreted cautiously, because the active sham subgroup comprised only six eyes. The observed differences do not establish that LLLT should preferentially be used during the active phase of thyroid eye disease, nor do they demonstrate that early treatment preserves meibomian gland function. These hypotheses require confirmation in larger prospective studies with adequate representation of patients with active and inactive disease.

### 2.5. Association Between Clinical Activity Score and OSDI

**Figure 7:**
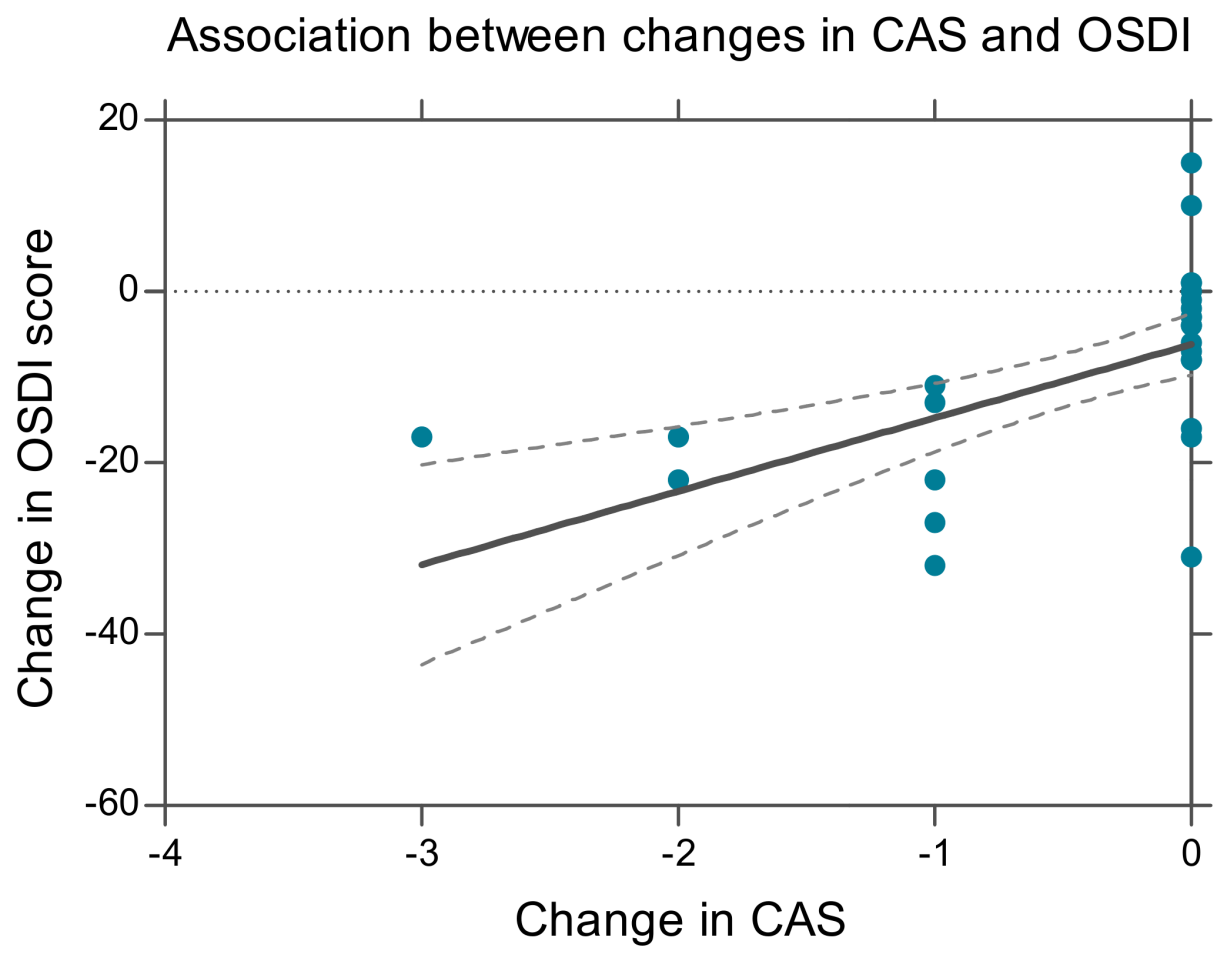
Association between change in Clinical Activity Score (CAS) and change in OSDI from T0 to T5. Changes were calculated as T5 minus T0. The fitted line is descriptive; inference is based on Spearman rank correlation (ρ = 0.639; Holm-adjusted *p* < 0.001).

CAS and OSDI were positively correlated at baseline (Spearman ρ = 0.547; Holm-adjusted *p* < 0.001) and at T5 (Spearman ρ = 0.428; Holm-adjusted *p* = 0.003). Changes from T0 to T5 were also positively correlated (Spearman ρ = 0.639; Holm-adjusted *p* < 0.001), indicating that larger reductions in clinical activity tended to accompany larger reductions in symptom burden (Figure 7). Pearson sensitivity analyses were directionally concordant (r = 0.397, 0.432, and 0.514, respectively). Because these correlations encompass both the sham-controlled group (n = 16) and the LLLT-treated group (n = 30), part of the association between changes in CAS and OSDI reflects the overall between-group therapeutic response, and the analysis should be interpreted as exploratory.

### 2.6. Representative Clinical Findings

**Figure 8:**
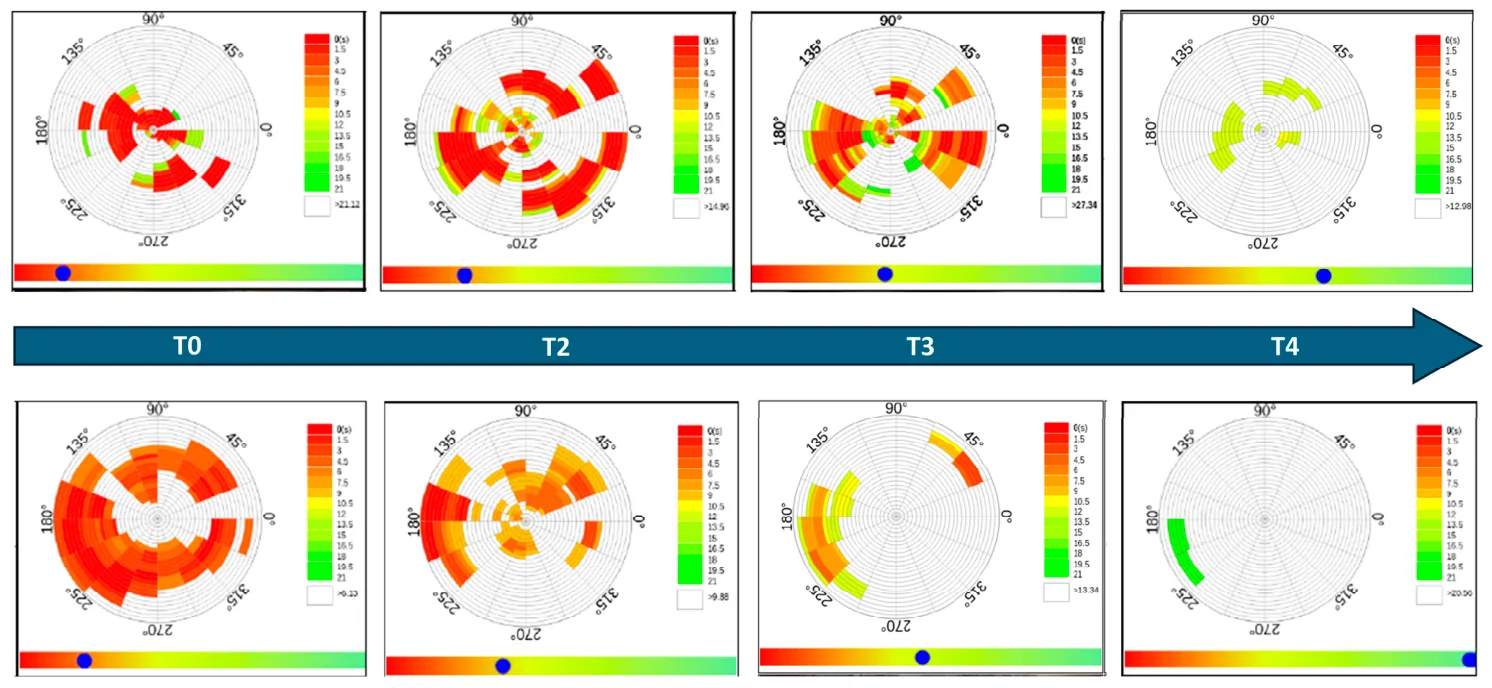
Representative serial non-invasive break-up maps from treated eyes. Color-coded polar maps display the local break-up time in each sector (red = early break-up; green = late break-up); the horizontal bar below each map indicates the overall tear film stability index. Successive examinations, in the direction of the arrow, show progressive migration from red-dominant, early and spatially fragmented break-up towards yellow-to-green, later and more homogeneous break-up.

**Figure 9:**
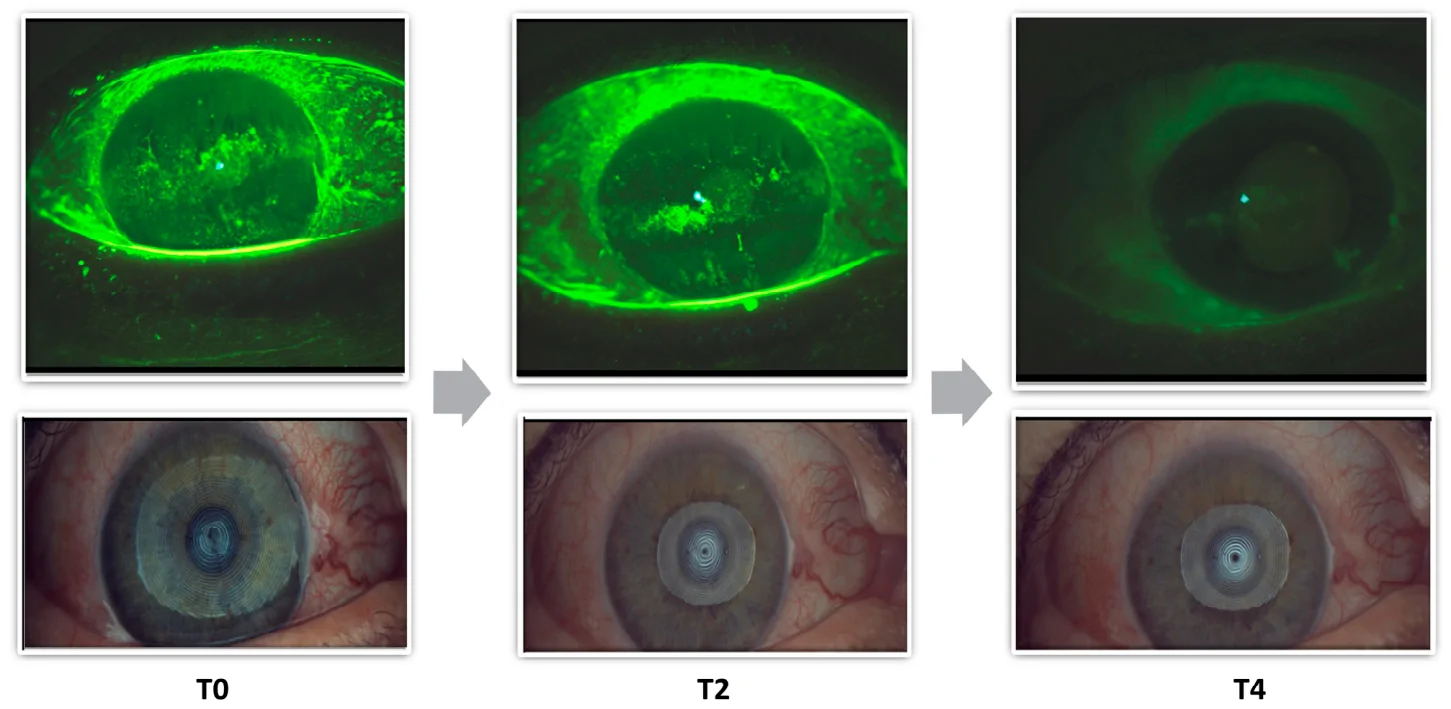
Representative clinical response in a treated eye. Upper row: slit lamp photographs with fluorescein and cobalt blue illumination at baseline (**left**), after 2 treatment sessions (**centre**), and 1 month after the last treatment (**right**), showing progressive resolution of confluent punctate epithelial staining. Lower row: corresponding diffuse illumination photographs of the same eye.

**Figure 10:**
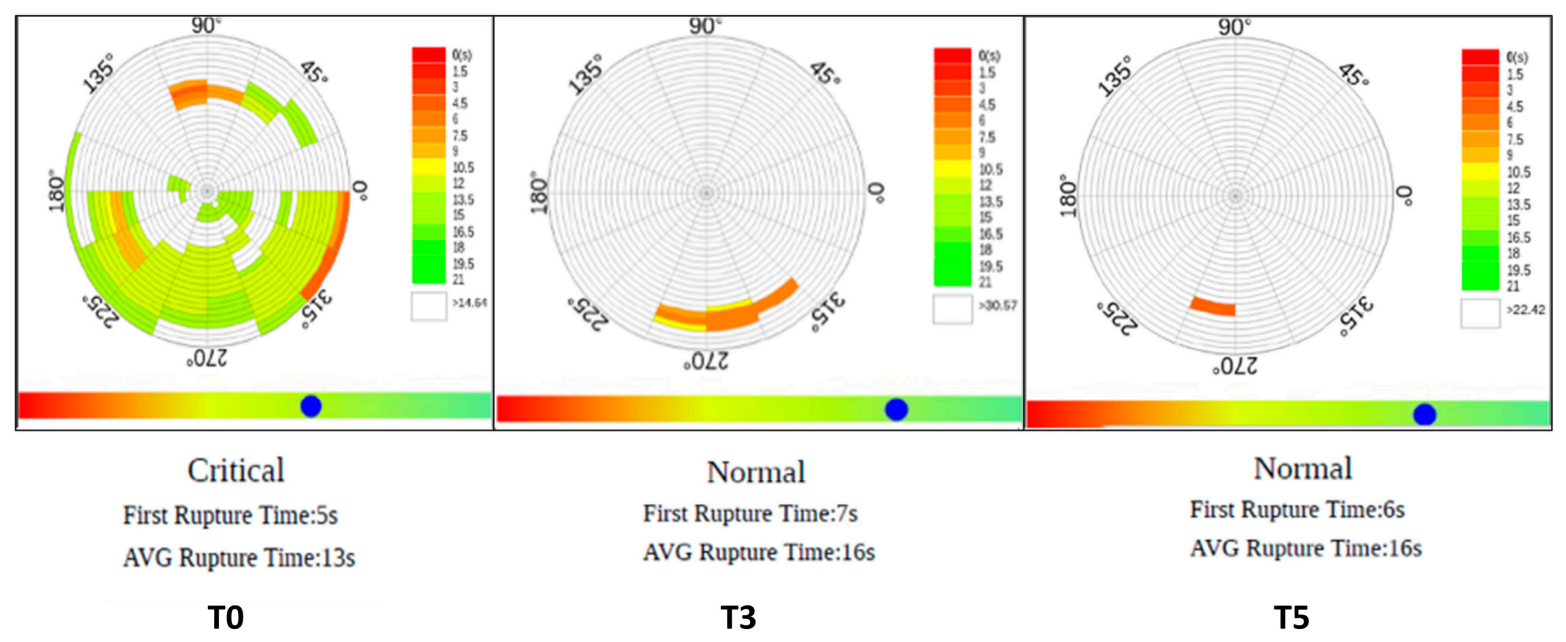
Serial automated tear film break-up reports from treated eyes. Successive examinations document the transition from the critical to the normal range on T0 (baseline), T3 (3 weeks after T0; last session treatment) and T5 (2 months after T3).

Representative examples of the qualitative changes observed in treated eyes are shown in Figure 8, Figure 9 and Figure 10. Serial color-coded polar break-up maps illustrate the progressive reorganization of the tear film, with a shift from early, widespread break-up (red-coded sectors) at baseline towards later and more spatially homogeneous break-up (yellow-to-green sectors) at the final visit (Figure 8). Corresponding slit lamp photographs with fluorescein show a marked reduction in punctate epithelial staining over the treatment course (Figure 9). Serial automated tear film break-up reports from a single treated eye document the transition of both first and average break-up times from the critical to the normal reference range (Figure 10). These images are illustrative of individual responders and are not intended to substitute for the group-level analyses reported above; notably, the group-level corneal staining analysis did not reach statistical significance, which underlines the limited sensitivity of semi-quantitative grading scales to focal epithelial change.

## 3. Discussion

This randomized, double-masked, sham-controlled trial shows that, in patients with TED-associated DED refractory to maximal conservative therapy, LLLT produces a significant and sustained reduction in symptom burden together with an objective reduction in tear hyperosmolarity, while leaving break-up time, aqueous secretion, gland morphology and lipid layer essentially unchanged over two months. The effect is therefore selective rather than global, and in exploratory analyses its magnitude appeared to vary with the inflammatory activity of the underlying orbitopathy.

The described pattern is informative in itself. Ocular surface disease in TED is not a single entity but the combination of exposure-related evaporative loss, meibomian gland dysfunction, tear film instability, hyperosmolar stress and inflammatory epithelial damage [7,8,11]. A therapy that acts on mitochondrial metabolism and on the resolution of inflammation would be expected to modify some of these components and not others, and that is precisely what was observed.

### 3.1. Symptomatic Burden and Disease Activity

Symptomatic burden in TED tracks inflammatory activity closely. In a cross-sectional study from the University of Pisa, patients with active orbitopathy had a significantly higher frequency of pathological OSDI scores than those with inactive disease, and multivariate analysis identified CAS as an independent predictor of OSDI, whereas age, sex and disease duration were not [16]. Eckstein et al. [17] found higher OSDI values in TED patients than in controls, Xu et al. [18] reported significantly higher scores in active than in inactive disease, and Allam et al. [19] described median OSDI values of 40.0 ± 5.80 in active versus 26.5 ± 5.10 in inactive TED. Riguetto et al. [20] similarly distinguished the inflammatory from the non-inflammatory phase on the basis of ocular surface involvement, and dry eye is well documented even in mild orbitopathy [21].

The present data reproduce this relationship and extend it to the therapeutic domain. CAS and OSDI were positively correlated at baseline and at the end of follow-up, and their changes over time were strongly correlated. At the end of treatment (T3), the baseline-adjusted between-group difference in OSDI was −7.49 points (95% CI, −11.66 to −3.32; *p* < 0.001), favoring LLLT. Although the unadjusted between-group difference in change from baseline exceeded the 8-point threshold used for sample size planning, the adjusted point estimate was slightly below this threshold. This distinction highlights that statistical significance does not necessarily imply that the adjusted treatment effect estimate reaches a clinically meaningful threshold. At the additional follow-up assessments, the adjusted LLLT-minus-sham differences were −11.97 points at T4 and −16.86 points at T5, suggesting that the symptomatic benefit persisted and increased after treatment cessation. Both estimates exceeded the 8-point threshold used for sample size planning. However, these follow-up assessments were not specifically powered in the original sample size calculation and should therefore be interpreted accordingly. The treatment effect estimates were similar across the analytical approaches examined, including additional adjustment for observed predictors of missing T5 OSDI. However, this consistency does not exclude bias due to missing outcomes.

### 3.2. Tear Osmolarity

Tear hyperosmolarity is the central amplifying mechanism of the vicious circle of DED, driving epithelial stress, apoptosis and inflammatory signaling. The reduction of approximately 11 mOsm/L at T4 and 14 mOsm/L at T5 in treated eyes is therefore significant, as it moves the ocular surface from a sustained hyperosmolar state towards the physiological range which can be correlated to the subjective symptoms.

Importantly, osmolarity was one of the few objective measures that showed a response. This may be because it reflects overall tear film quality, distribution, and evaporative loss, rather than measuring only structural changes.

### 3.3. Tear Film Stability and the Lipid Layer

Tear film stability, assessed by NIBUT, is reduced in TED compared with healthy controls and is further shortened in active disease, mirroring the severity of corneal staining and of symptoms [8,22]. Qualitative alterations of the tear film in active orbitopathy were described early on by Nowak et al. [23], and characteristic break-up patterns have since been mapped in this population [22]. This instability is multifactorial, arising from widening of the palpebral fissure, increased evaporation and reduction of both membrane-associated and secretory mucins under inflammatory conditions [11]. Improvements in break-up time have been reported after LLLT-based treatment in evaporative DED: Pérez-Silguero et al. [24] described sustained gains after combined intense pulsed light and LLLT, Giannaccare et al. [25] reported improved tear film parameters after serial sessions of a home LLLT device, and Park et al. [26] found significant benefit in a randomized observer-masked trial.

In the present cohort neither NIBUT nor FBUT differed between groups after multiplicity adjustment. Two explanations deserve consideration. First, in TED the dominant determinants of break-up time are mechanical factors such as palpebral aperture, lagophthalmos and blinking pattern and these are unaffected by periocular light. Second, all participants were maintained on intensive lubrication and bedtime ointment, which by design supports tear film stability in both arms and compresses the difference measurable between them. As stated above, there were no significant differences in the lipid layer in either the sham group or the LLLT group, which may, to some extent, explain the absence of subsequent changes in tear film stability.

### 3.4. Meibomian Gland Morphology

Meibomian gland dysfunction is a major contributor to ocular surface deterioration in TED, with obstructive disease reaching very high prevalence in symptomatic patients and greater gland loss in active than in inactive disease [27]. Inoue et al. documented significantly higher meiboscores in TED patients than in controls, with dropout concentrated in the central third of the upper eyelid, a distribution attributed to mechanical stress from levator hypertrophy and fibrosis [27]. Aghaei et al. found greater upper-lid and total gland loss in moderate-to-severe than in mild disease [28].

In the present study neither upper- nor lower-eyelid meibography changed significantly after multiplicity adjustment, although the lower-eyelid estimate at T5 favored LLLT (−4.39) at nominal significance. This finding is not unexpected. Meibomian gland dropout in TED reflects established structural atrophy accumulated over years, and there is no reason to expect anatomical regeneration within a two-month window.

### 3.5. Aqueous Secretion

Aqueous tear production is also affected in TED, with lower Schirmer values reported than in healthy controls, although with considerable variability across disease stages and break-up patterns [7,22]. No between-group difference in Schirmer values was demonstrated here. The slightly lower numbers seen in treated eyes likely point to decreased reflex tearing secondary to improved surface comfort rather than as a decline in lacrimal function. This interpretation remains speculative in the absence of a reflex-versus-basal secretion measurement.

### 3.6. Corneal Staining

Corneal fluorescein staining is increased in active disease and reflects epithelial compromise [19]. The adjusted probability of staining was lower with LLLT at both endpoints, but the confidence intervals were wide and no significant difference was demonstrated. The illustrative cases in Figure 9 show unmistakable focal improvement in individual eyes, which the Oxford grading did not translate into a group-level effect. Semi-quantitative scales reduce complex and uneven epithelial changes to a few categories, which can make it difficult to detect small improvements. In future trials, measuring the stained area automatically would provide a more accurate way to assess change.

### 3.7. Acute Versus Chronic Ocular Surface Injury: Why Photobiomodulation Behaves Differently in Cataract Surgery and in Thyroid Eye Disease

An informative comparison can be drawn between the present findings and those obtained with the same technology in the perioperative setting of cataract surgery, which we and others have investigated. In a prospective, double-masked, randomized sham-controlled trial in 89 patients undergoing phacoemulsification, two perioperative LLLT sessions produced significant improvements in OSDI (26.62 ± 15.42 at baseline to 15.53 ± 12.04 at one week and 13.36 ± 11.69 at one month), in break-up time (5.76 ± 3.99 to 8.38 ± 4.53 s) and in tear osmolarity (300.69 ± 14.19 to 296.11 ± 12.30 mOsm/L) within a single postoperative month [29]. Giannaccare et al. observed the same pattern in 131 patients, with significantly lower OSDI scores, higher NIBUT values and lower meiboscores in LLLT-treated eyes after two perioperative sessions [30]. In a subsequent trial integrating tear biomarkers, LLLT-treated eyes showed a far higher rate of clinical improvement than sham-treated eyes (44.2% vs. 4.4%; *p* < 0.0001), with increases in reparative and neurotrophic mediators (GDF-15, PDGF-CC, β-NGF) and a biomarker profile indicating resolution of inflammation, whereas sham-treated eyes showed persistent inflammatory activity [31].

These studies and the present one differ in the nature of the insult rather than in the intervention. Cataract surgery imposes an acute, time-limited, iatrogenic injury—corneal incisions with transection of sub-basal nerves, intraoperative light and irrigation exposure, and a transient preservative and inflammatory load from postoperative drops—upon an ocular surface that is otherwise structurally intact and served by a lacrimal functional unit with preserved reparative capacity. Under these conditions, photobiomodulation affects a temporary process whose natural trajectory is already towards resolution. As a result, its effects can appear quickly, be quite large, and be seen in both subjective and objective measures within a few weeks.

In TED the situation is fundamentally different. The ocular surface insult is not a discrete event but the continuous downstream consequence of an ongoing, systemically driven autoimmune process. Proptosis and eyelid retraction widen the palpebral aperture and increase evaporative loss; lagophthalmos and altered blink dynamics perpetuate exposure; muscles and orbital tissue remodeling produces sustained mechanical stress on the meibomian glands; and lacrimal gland involvement adds an aqueous-deficient component. None of these described factors is modifiable by periocular light. Photobiomodulation addresses only downstream effects—such as peri-glandular inflammation, meibum quality, lipid layer structure, and epithelial homeostasis. It leaves the root cause of the disease untreated, while established glandular atrophy restricts the potential for functional recovery.

Two additional observations support this interpretation. First, the treatment showed a distinct temporal pattern compared to acute post-cataract setting: while post-cataract surgery benefits typically peak within the first month before plateauing, the adjusted between-group OSDI difference in this study continued to widen from T4 (−11.97) to T5 (−16.86) after treatment ceased, which suggests a cumulative modulation of a chronic process rather than the rapid correction of a transient one. Second, baseline inflammatory activity strongly modified the treatment benefit, contrasting with surgical settings where baseline inflammatory states are uniformly low.

The practical consequence is that therapeutic expectations must be framed differently in the two contexts. In perioperative care, LLLT can be offered as prophylaxis with a predictable, short-term and largely uniform return. In TED it should be positioned as an adjunctive and repeatable modality within a multimodal strategy, with explicit counselling that the benefit is likely to be symptomatic and progressive rather than immediate and structural, that it is greatest in eyes with active inflammation, and that it remains contingent on control of the underlying orbitopathy. It also follows that the response to LLLT in TED is intrinsically harder to predict at the individual level than in the postoperative setting, and that predictive models of the kind developed for cataract surgery [31] will require disease-specific recalibration before they can be transferred to autoimmune ocular surface disease.

### 3.8. Clinical Implications

Taken together, these findings support a role for LLLT as a safe adjunctive option in TED-associated DED, in patients with persistent symptoms despite maximal lubrication. Two hypotheses arising from the exploratory analyses deserve testing in future trials. First, because the effect on symptoms appeared larger in active disease, LLLT may be most useful during the inflammatory phase, in parallel with systemic management of the orbitopathy, rather than reserved for chronic disease. Second, because no structural improvement in gland morphology was demonstrable, early intervention, before irreversible dropout accumulates, might better preserve the functional reserve on which the treatment appears to act.

### 3.9. Strengths

The strengths of this trial include its randomized, double-masked, sham-controlled design, assessment by a single masked examiner under standardized conditions, and longitudinal evaluation extending two months beyond treatment. The originally specified primary OSDI assessment was at T3, while T4 and T5 provided additional information on the evolution of the treatment response during follow-up. Effect estimates are reported with confidence intervals throughout, and the T4 and T5 estimates were subjected to complete-case and ANCOVA sensitivity analyses that produced closely concordant results.

### 3.10. Limitations

Several limitations must be acknowledged. The sample was modest and the groups unequal: the 2:1 allocation left only 16 sham eyes, and the active sham subgroup comprised six eyes, so the exploratory subgroup estimates are imprecise and should be regarded as hypothesis-generating despite the significant interaction term. OSDI availability decreased to 78.3% at T5 (22/30 LLLT and 14/16 sham), owing to the exclusion of incomplete questionnaires rather than missed visits. Missing T5 OSDI was associated with age, baseline CAS, and baseline quality-of-life score in exploratory analyses. Additional adjustment for these characteristics produced treatment effect estimates similar to those from the original model. However, this analysis does not establish the missing-at-random assumption or exclude bias arising from unobserved determinants of missingness.

A limitation specific to this cohort concerns background therapy. All participants were on maximal conventional treatment, that was deliberately maintained unchanged in both arms throughout the study, consisting of intensive preservative-free artificial tears during the day, a lubricating ointment at bedtime and daily periocular hygiene. This was ethically necessary in a symptomatic refractory population, but it sets a high therapeutic floor: lubrication and lid hygiene themselves support tear film stability, lipid delivery and epithelial integrity, and they may therefore attenuate the incremental effect attributable to LLLT, particularly for evaporative measures such as break-up time. The negative secondary outcomes should be read in this light and not as evidence that LLLT is inactive on those parameters. Conversely, the same design feature strengthens the interpretation of the primary outcome, since the observed symptomatic benefit is an effect obtained over and above maximal conservative management. Adherence to background therapy was reviewed verbally rather than measured objectively, which introduces a further source of variability.

The primary outcome is patient-reported and, although both participants and examiners were masked and the sham procedure was sensorially equivalent, symptom endpoints remain more susceptible to expectation effects than objective measures; the concordant osmolarity findings partially, but not completely, mitigate this concern. The study was powered for the originally specified OSDI comparison between baseline and T3, not for the additional T4 and T5 follow-up assessments. Consequently, the later estimates should be interpreted as additional evidence on the evolution of the treatment response rather than as independently powered primary comparisons. Additionally, only one eye per patient was analyzed, which reduces the information available but avoids inter-eye correlation. Follow-up extended only two months beyond the last session, so the durability of the effect and the optimal retreatment interval remain unknown—a question made more relevant by the observation that the between-group difference was still increasing at the last visit. Mechanistic inference is indirect: no tear cytokine or biomarker profiling was performed in this cohort, and disease activity was captured only by CAS, without TRAb dynamics or orbital imaging correlates. Lagophthalmos, palpebral aperture and blink completeness—plausibly the dominant determinants of the negative break-up time results—were not quantified.

An additional limitation specific to the application of LLLT in thyroid eye disease concerns the potential influence of exophthalmos on light delivery. Although the manufacturer’s recommended distance between the light source and the closed eyelids was maintained, irradiance and radiant exposure at the ocular surface were not directly measured or adjusted according to the degree of proptosis. A forehead spacer was required for comfort in only one participant receiving active LLLT, and its potential influence on the delivered dose was not quantified. As this study represents an early investigation of LLLT in this specific clinical context, treatment parameters were based on the manufacturer’s standardized protocol rather than individualized according to orbital anatomy. Future studies should investigate the relationship between the degree of exophthalmos, device-to-tissue distance, irradiance, and delivered radiant exposure, with the aim of optimizing and personalizing treatment parameters for patients with varying degrees of proptosis.

Finally, the cohort was single-country, predominantly female and restricted to mild-to-moderate TED, so the findings should not be extrapolated to sight-threatening disease or to populations with different treatment backgrounds.

## 4. Materials and Methods

### 4.1. Study Design and Participants

This prospective, randomized, double-masked, sham-controlled clinical trial was conducted at the Ophthalmology Clinic of the Cai Ferate Clinical Hospital and the Grigore T. Popa University of Medicine and Pharmacy, Iasi, Romania, and at the Optissima Clinic, Bucharest, Romania. The study protocol was approved by the local Institutional Ethics Committee (approval no. 598/20.05.2025) and prospectively registered on ClinicalTrials.gov (NCT07332988) on 12 January 2026. The Ethics Committee-approved protocol designated the Ocular Surface Disease Index (OSDI) score as the primary outcome and based the sample size calculation on the change in OSDI from baseline (T0) to three weeks (T3). The ClinicalTrials.gov registration listed tear film stability, assessed using non-invasive tear break-up time (NIBUT) and fluorescein tear break-up time (FBUT) at three months, as the primary outcome, with repeated measures analysis of variance (ANOVA) or the Friedman test specified for longitudinal comparisons. In the present study, the primary and secondary outcomes were analyzed according to the Ethics Committee-approved protocol, with OSDI as the primary outcome and NIBUT and FBUT as secondary outcomes. For longitudinal analyses, baseline-adjusted mixed effects models were used instead of the originally registered repeated measures tests to account for uncompleted OSDI questionaries at T4 and T5 and incorporate all available post-baseline observations. All participants provided written informed consent before enrolment. All procedures conformed to the tenets of the Declaration of Helsinki and its subsequent revisions.

Consecutive patients aged 18 years or older with DED associated with TED, who remained symptomatic despite maximal conventional ocular surface therapy, were screened and recruited between 13 January and 30 March 2026, with the first participant enrolled on 13 January 2026. Following enrolment, each participant underwent four treatment sessions administered once weekly. Upon completion of the treatment regimen, participants entered a two-month follow-up period, with assessments performed 1 month after the final treatment session (T4) and two months after the last treatment session (T5).

Exclusion criteria were (i) pregnancy or breastfeeding; (ii) uncontrolled systemic disease other than Graves’ disease; (iii) systemic corticosteroid use within the preceding 6 months; (iv) ocular or orbital surgery within the preceding 6 months; (v) contact lens wear within the preceding week; and (vi) active ocular infection or allergy.

DED was diagnosed using a TFOS DEWS III diagnostic approach, requiring the presence of dry eye symptoms assessed with the OSDI (>13, on a scale from 0 to 100) together with at least one objective sign of tear film or ocular surface homeostasis disturbance, defined as NIBUT < 10 s, tear hyperosmolarity (osmolarity ≥ 308 mOsm/L), or >5 corneal fluorescein staining spots.

TED was diagnosed according to EUGOGO recommendations, on the basis of autoimmune thyroid disease (elevated TSH receptor antibodies) together with orbital findings such as eyelid retraction, proptosis, restricted ocular motility or restrictive diplopia, or inflammatory orbital changes [4,5]. Disease activity was assessed with the CAS, a score ≥3 defining active disease. Severity was classified as mild, moderate-to-severe or sight-threatening according to EUGOGO criteria; the cohort comprised patients with mild to moderate disease, in both active and inactive phases. The quality of life of the patients was assessed using the EUGOGO disease-specific Quality of life Questionnaire (GO-QoL). All examinations were performed by the same masked examiner under standardized environmental conditions.

One eye per patient was included, and all analyses were performed at the eye level, so that the number of eyes equals the number of patients throughout.

### 4.2. Concomitant Ocular Surface Therapy

All participants had been receiving maximal conventional ocular surface therapy for at least one month before enrolment. This treatment regimen was maintained unchanged in both study arms throughout the study period. The treatment consisted of alternating administration of preservative-free ophthalmic drops containing 3% trehalose and 0.15% sodium hyaluronate and an ophthalmic gel containing 3% trehalose, 0.15% sodium hyaluronate, and 0.25% carbomer, at a combined frequency of 6 applications daily. Additionally, a lubricating ophthalmic ointment containing vitamin A (retinyl palmitate, 250 IU/g) was applied twice daily. All participants also performed daily eyelid hygiene, including warm compresses followed by gentle lid margin cleansing.

For the purposes of this study, refractory dry eye disease was defined as the persistence of ocular surface symptoms and clinical signs despite adherence to this optimized conventional treatment regimen for at least one month, with an Ocular Surface Disease Index (OSDI) score > 13 at enrolment.

Participants were instructed not to modify the type, brand or frequency of their lubricant therapy, and adherence was reviewed verbally at every visit.

Continuation of background therapy was considered ethically necessary in a symptomatic, refractory cohort. It also defines the clinical question addressed by this trial, which is not whether LLLT is superior to no treatment, but whether it adds measurable benefit on top of maximal conservative management. Systemic treatment for Graves’ disease was managed by the referring endocrinologist and was not modified for study purposes.

### 4.3. Randomization and Masking

The participants were first assessed for eligibility, continued by the random selection of the study eye, after which they were randomly assigned in a 2:1 ratio to either the active LLLT group or the sham control group. Although only one eye per patient was examined, the treatment was nevertheless administered to both eyes. The allocation sequence was computer-generated (www.sealedenvelope.com) and prepared by an external statistician who had no clinical contact with participants. Allocation concealment was ensured through sequentially numbered, opaque, sealed envelopes. The treatment operator opened the corresponding envelope, implemented the assigned intervention, and administered either active LLLT or sham treatment according to the randomization sequence. Because the operator was responsible for delivering the intervention, they were not masked to treatment allocation; however, they were not involved in clinical outcome assessments. Both participants and the investigators responsible for clinical evaluation remained unaware of group assignment throughout the study period. All clinical assessments were performed independently by the same masked examiner. In both groups, the treatment procedure was identical in terms of device appearance, positioning, and session duration, with the only difference being the light output delivered.

### 4.4. Ophthalmic Evaluation

All patients underwent a comprehensive ophthalmic examination including best-corrected visual acuity, intraocular pressure, exophthalmometry and ocular surface evaluation. Symptoms were quantified with the Ocular Surface Disease Index (OSDI), which was the primary outcome measure. A disease-specific quality-of-life score was recorded at baseline.

Objective tear film assessment comprised non-invasive break-up time (NIBUT), fluorescein break-up time (FBUT), corneal fluorescein staining graded on the Oxford scale, the Schirmer I test without topical anesthesia, meibomian gland morphology and tear osmolarity. NIBUT, lipid layer grade and meibography (meiboscale grading of upper and lower eyelids in relation to the degree of gland loss) were obtained with the DEA 520 automated ocular surface analyzer (Shanghai MediWorks Precision Instruments Co., Ltd., Shanghai, China), which also generates color-coded polar break-up maps of the tear film. Tear osmolarity was measured with the TearLab Osmolarity System. Diagnostic thresholds followed TFOS DEWS III recommendations [32].

To limit measurement bias, all tests were performed in the same order, by the same masked examiner, in a room with controlled temperature and humidity, with the least invasive tests performed first.

### 4.5. Treatment Protocol

The active group received periocular LLLT with the Eye-light^®^ device (Espansione Group Ltd., Funo, Italy), an LED mask emitting 630 nm red light, with an irradiance of 35 mW/${cm}^{2}$, with a total delivery of 32 J/${cm}^{2}$. The light was delivered at a distance of 5–10 mm from the closed eyelids for 15 min per session. Sessions were carried out at baseline (T0) and weekly over a three weeks period (T1, T2 and T3).

The sham group underwent an identical procedure, using the same device, session duration, and treatment operator, but with light emission reduced to less than 30% of the therapeutic output. The same device and sham configuration have previously been used in randomized sham-controlled clinical trials investigating ocular surface outcomes following cataract surgery [29,30,31], and dry age-related macular degeneration [33]. In these studies, the sham intervention was not associated with clinically meaningful improvements in the evaluated clinical parameters. Consistent with these findings, OSDI scores remained relatively stable in the sham group of the present study throughout follow-up. Taken together, previous clinical experience and the absence of a meaningful clinical response in our sham group support the use of this reduced output configuration as a clinically inactive comparator for the assessed outcomes. Nevertheless, although its therapeutic inactivity is supported by the available clinical evidence, complete biological inertness cannot be definitively established, as the biological effects of the reduced light exposure were not directly investigated.

To ensure effective masking, both active and sham masks had a similar visual appearance, and the procedures were identical in terms of device appearance, positioning, and session duration, with the difference being the light output delivered.

In one participant in the active LLLT group with more pronounced exophthalmos, a forehead spacer was used to improve comfort by increasing the distance between the device and the periocular area. Although the manufacturer’s recommended distance of 5–10 mm was maintained, irradiance and radiant exposure at the ocular surface were not directly measured. Therefore, a potential influence of the spacer on the delivered dose cannot be entirely excluded. This limitation should be considered when interpreting the treatment exposure, particularly in patients with anatomical variations associated with exophthalmos.

### 4.6. Outcomes and Clinical Assessments

Ocular surface assessments were performed at six predefined time points: baseline, before the first treatment session (T0); weekly during treatment (T1, T2, and T3); and at one month (T4) and two months (T5) after the last treatment session at T3. The primary outcome was the Ocular Surface Disease Index (OSDI) with the change from baseline to T3 representing the originally specified primary assessment. T4 and T5 were additional follow-up assessments intended to characterize the persistence and further evolution of the treatment response beyond the end of treatment. Secondary outcomes included NIBUT, FBUT, the Schirmer test, tear osmolarity, upper- and lower-eyelid meibography, corneal staining, and lipid layer grade.

OSDI, NIBUT, FBUT, and lipid layer grade were evaluated at all visits (T0–T5). Tear osmolarity, meibography, the Schirmer I test and corneal fluorescein staining were assessed at T0, T4 and T5. For the additional follow-up assessments, baseline-adjusted between-group differences were also estimated at T4 and T5 to characterize the evolution of the treatment response after completion of treatment.

Complete OSDI questionnaires were available for all participants from baseline (T0) through T3 (30 in the LLLT group and 16 in the sham group at each assessment). At T4, 29/30 (96.7%) questionnaires in the LLLT group and 15/16 (93.8%) in the sham group met the completeness criteria, yielding 44 questionnaires overall. At T5, 22/30 (73.3%) questionnaires in the LLLT group and 14/16 (87.5%) in the sham group met these criteria, yielding 36 questionnaires overall. Accordingly, one questionnaire in each group was excluded at T4, and 8 (LLLT) and 2 (sham) were excluded at T5; all participants in the analysis population attended these visits. Only questionnaires with all required items completed and the same number of valid responses maintained between baseline and the respective follow-up assessment were included in the statistical analyses. Some participants left items concerning environmental exposure, including air-conditioned and dry air environments, unanswered; therefore, questionnaires that did not meet these completeness criteria were excluded from the corresponding OSDI analyses. These exclusions should be distinguished from discontinuation of the intervention or loss to follow-up.

### 4.7. Sample Size

The sample size was determined a priori on the primary outcome, defined as the between-group difference in change in OSDI score from baseline (T0) to the last treatment session performed 3 weeks after (T3). The calculation assumed a clinically meaningful between-group difference of 8 OSDI points, a standard deviation of 10 points, a two-sided significance level of 0.05, 90% statistical power, and a 2:1 allocation ratio. Based on these assumptions, the target sample size was approximately 46 evaluable participants. Allowing for an anticipated 10% dropout rate, the planned recruitment target was approximately 51 participants. The sample size calculation was based on the T0–T3 comparison and was not specifically powered for the additional follow-up assessments at T4 and T5. The 8-point difference was used as the clinically meaningful effect size for sample size planning and was not considered a statistical significance threshold.

### 4.8. Safety and Compliance

Of the 51 randomized participants, two in the LLLT group discontinued treatment for personal reasons, and three were lost to follow-up (one LLLT and two sham), as de-scribed in Section 2.1 and Figure 1; none of these withdrawals was related to an adverse event.

In one participant allocated to the active LLLT group who presented with more pronounced exophthalmos, a greater perception of local warmth was reported during treatment. This was managed by inserting a forehead spacer to increase the distance between the device and the periocular area. This transient discomfort did not lead to discontinuation and was not considered an adverse event; no adverse events were recorded, supporting the overall safety and tolerability of the intervention.

The forehead spacer was used for comfort in this participant only and was not required in the sham group. Although the manufacturer’s recommended distance between the light source and the treated area was maintained, irradiance and radiant exposure at the ocular surface were not directly measured; therefore, a potential influence of the spacer on the delivered dose cannot be entirely excluded. Given the anatomical variations associated with exophthalmos, future studies should consider adapting treatment parameters according to the degree of proptosis and directly measuring irradiance at the ocular surface. These measures could improve dose estimation, support individualized dose optimization, and enhance treatment standardization in patients with varying degrees of exophthalmos.

### 4.9. Statistical Analysis

Analyses were performed at the eye level using 46 observations (30 assigned to LLLT and 16 to sham). The analysis population was a modified intention-to-treat population, defined as all randomized participants with baseline data and at least one evaluable post-baseline OSDI assessment, analyzed according to their allocated group. Five randomized participants (three LLLT, two sham) without any evaluable post-baseline assessment were excluded (Section 2.1). Baseline disease activity was defined as active for CAS ≥ 3 and inactive for CAS < 3. Continuous variables are presented as mean (standard deviation) or median (interquartile range), as appropriate, and categorical variables as number (percentage). Standardized mean differences (SMDs) were used to quantify baseline imbalance.

Only OSDI questionnaires that were fully completed with respect to all required items and for which the same number of valid responses was maintained between the baseline assessment and subsequent follow-up assessments were included in the statistical analysis. For longitudinal comparisons, the same number of valid responses was required at baseline and at the relevant follow-up assessment to ensure comparability of OSDI scores over time. The originally specified primary outcome was the between-group difference in change in OSDI score from baseline (T0) to the end-of-treatment assessment (T3). A baseline-adjusted linear mixed effects model was used to estimate treatment differences over time, with fixed effects for treatment, categorical visit, the treatment-by-visit interaction, baseline OSDI, and the baseline OSDI-by-visit interaction, and a random intercept to account for repeated observations within each participant. The adjusted LLLT-minus-sham contrast at T3 was used to evaluate the primary outcome. Additional baseline-adjusted contrasts at T4 and T5 were examined to characterize the treatment response during follow-up. These follow-up analyses were additional to the original sample size planning and were interpreted accordingly. Model-based estimates and 95% confidence intervals (CIs) are reported. The additional T4 and T5 contrasts were adjusted for multiplicity using the Holm procedure. Robustness was assessed using a complete-case mixed effects model and separate baseline-adjusted ANCOVA models at the additional follow-up assessments.

Continuous secondary outcomes were analyzed within the same baseline-adjusted mixed model framework when longitudinal data were available. Outcome-specific residual diagnostics were reviewed, and log-transformed and influence analyses were used as sensitivity analyses where distributional departures or influential observations were evident. Tear osmolarity and meibography were analyzed at T0, T4 and T5, with upper- and lower-eyelid meibography modelled separately. T4 and T5 contrasts were Holm-adjusted within each outcome. Secondary outcome analyses were considered supportive, and multiplicity adjustment was applied across the two endpoint contrasts within each outcome rather than across all secondary outcomes.

Corneal staining was defined as any grade greater than zero. Observed proportions with Wilson 95% CIs are reported across T0–T5. Because the longitudinal binary model was unstable, primary between-group inference used separate baseline-adjusted logistic regression models at T4 and T5, from which standardized probabilities, risk differences, odds ratios (ORs) and 95% CIs are reported.

Effect modification by baseline disease activity was explored for OSDI using a model containing the treatment-by-visit-by-activity interaction with baseline adjustment. Within-activity treatment contrasts, and the differences between activity-specific treatment effects, were estimated at T4 and T5.

Associations between CAS and OSDI were evaluated at T0 and T5 and for the change from T0 to T5. Spearman rank correlations were primary and Pearson correlations were sensitivity analyses; *p* values were Holm-adjusted across the three analyses. These analyses were exploratory and were performed across treatment groups without adjustment for treatment assignment. Tear film lipid layer thickness was assessed using the DEA 520 automated ocular surface analyzer (MediWorks, China) by interferometric pattern analysis. The lipid layer was classified into four grades according to the manufacturer’s grading criteria: Grade 1 (<30 nm), Grade 2 (30–60 nm), Grade 3 (60–80 nm), and Grade 4 (>80 nm). The resulting categorical data were used for between-group and within-group comparisons. The distribution of tear film lipid layer grades was compared between the sham and low-level light therapy (LLLT) groups at baseline (T0) and each follow-up assessment (T1–T5), as well as changes over time within each group. Lipid layer grade was treated as a categorical variable, and differences in grade distributions were assessed using the chi-squared test. All tests were two-sided with α = 0.05, and statistical significance was accepted for *p* ≤ 0.05. Analyses were performed in R version 4.3 (R Foundation for Statistical Computing, Vienna, Austria) and GraphPad Prism 5 (San Diego, CA, USA).

## 5. Conclusions

In patients with dry eye disease associated with thyroid eye disease who remained symptomatic despite intensive artificial tears, bedtime ointment and daily periocular hygiene, low-level light therapy produced a significant and progressively increasing reduction in symptom burden and an objective reduction in tear hyperosmolarity, without any treatment-related adverse events. No significant effect was demonstrable on break-up time, aqueous secretion, meibomian gland morphology or lipid layer grade within two months, and, in exploratory analyses, the symptomatic benefit appeared in eyes with active orbital inflammation.

The selective nature and slower pace of this response, when set against the rapid and broad improvement obtained with the same technology after cataract surgery, illustrate a general principle: photobiomodulation modifies the ocular surface consequences of injury most effectively when the injury is acute and self-limiting, and only partially when it is continuously renewed by an uncontrolled systemic process. In thyroid eye disease, LLLT should therefore be positioned as a safe adjunctive component of multimodal care, rather than as a substitute for control of the underlying orbitopathy. Larger and longer randomized trials, with balanced allocation, quantified eyelid biomechanics and mechanistic biomarker endpoints, are needed to confirm these findings and to define optimal patient selection and retreatment schedules.

## Figures and Tables

**Table 1:** Baseline characteristics of the two treatment groups.

Characteristic	Sham (*n* = 16)	LLLT (*n* = 30)	SMD	*p* Value
Age, median (years)	57	57	0.17	0.392
Female, *n* (%)	12 (75.0)	24 (80.0)	0.12	0.720
CAS, median	2	2	0.44	0.189
Active disease (CAS ≥ 3), *n* (%)	6 (37.5)	14 (46.7)	0.19	0.756
Quality-of-life score, median	70.5	34.0	0.82	0.030
OSDI, median	25.5	33.0	0.49	0.127
Primary gaze IOP, mean (SD), mmHg	18.4 (2.6)	15.8 (2.6)	1.00	0.003
Exophthalmometry, mean (SD), mm	22.8 (3.0)	20.8 (3.0)	0.66	0.041

CAS = Clinical Activity Score; IOP = intraocular pressure; LLLT = low-level light therapy; OSDI = Ocular Surface Disease Index; SD = standard deviation; SMD = standardized mean difference.

**Table 2:** Baseline-adjusted between-group differences (LLLT minus sham) for the secondary outcomes at T4 and T5.

Outcome	T4, LLLT—Sham (95% CI)	Holm-Adjusted *p*	T5, LLLT—Sham (95% CI)	Holm-Adjusted *p*
NIBUT, s	0.95 (−1.61 to 3.51)	0.464	−2.41 (−5.19 to 0.36)	0.176
FBUT, s	1.16 (0.11 to 2.21)	0.061	−0.63 (−1.76 to 0.50)	0.270
Schirmer I, mm	−2.17 (−5.49 to 1.14)	0.393	−0.87 (−4.36 to 2.61)	0.621
Tear osmolarity, mOsm/L	−10.91 (−18.96 to −2.87)	0.009	−13.81 (−22.55 to −5.06)	0.004
Upper-eyelid meibography	−1.67 (−4.71 to 1.36)	0.548	1.26 (−1.98 to 4.51)	0.548
Lower-eyelid meibography	−3.20 (−7.16 to 0.76)	0.110	−4.39 (−8.46 to −0.33)	0.070

CI = confidence interval; FBUT = fluorescein break-up time; LLLT = low-level light therapy; NIBUT = non-invasive break-up time. Confidence intervals are nominal 95% CIs; *p* values were multiplicity-adjusted with the Holm procedure across the two endpoint contrasts within each outcome.

## Data Availability

All data generated or analyzed during this study are included in the published article and its Supplementary Materials.

## Supplementary Materials

The following supporting information can be downloaded at https://www.mdpi.com/article/10.3390/ijms27219802/s1.

## Abbreviations

The following abbreviations are used in this manuscript:

ANCOVA	analysis of covariance
CAS	Clinical Activity Score
CI	confidence interval
DED	dry eye disease
EUGOGO	European Group on Graves’ Orbitopathy
FBUT	fluorescein break-up time
IOP	intraocular pressure
LED	light-emitting diode
LLG	lipid layer grade
LLLT	low-level light therapy
NIBUT	non-invasive break-up time
OR	odds ratio
OSDI	Ocular Surface Disease Index
SD	standard deviation
SMD	standardized mean difference
TED	thyroid eye disease
TFOS DEWS III	Tear Film and Ocular Surface Society Dry Eye Workshop III
TSHR	thyroid-stimulating hormone receptor
