# Supplementary material for: Low-Level Light Therapy for Dry Eye Disease in Thyroid Eye Disease: A Randomized, Double-Masked, Sham-Controlled Trial
Source: Int J Mol Sci. 2026 Oct 31;27(21):9802. doi: 10.3390/ijms27219802 (PMC13763280; doi:10.3390/ijms27219802)
Supplement: Supplementary file 1 [file ijms-27-09802-s001.zip › ijms-4631335-supplementary.pdf]

SUPPLEMENTARY MATERIAL

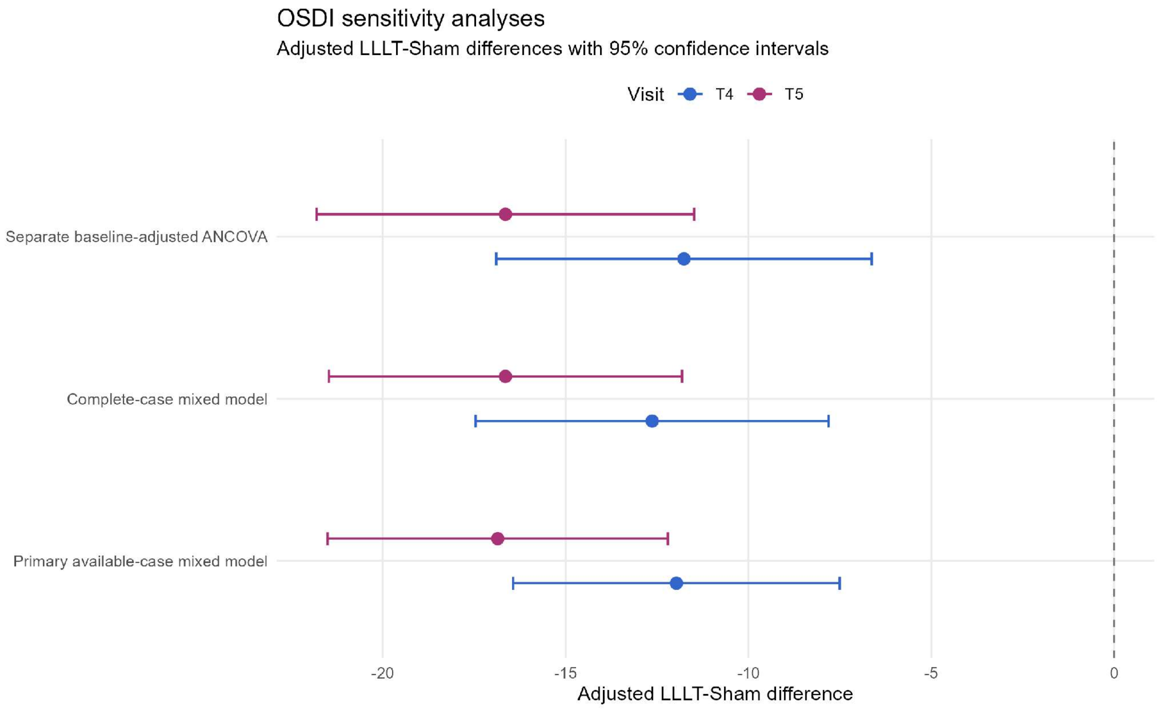

Supplementary Figure S1. Sensitivity analyses for the primary OSDI outcome. All analytical approaches produce clearly negative LLLT-minus-sham effects at T4 and T5.

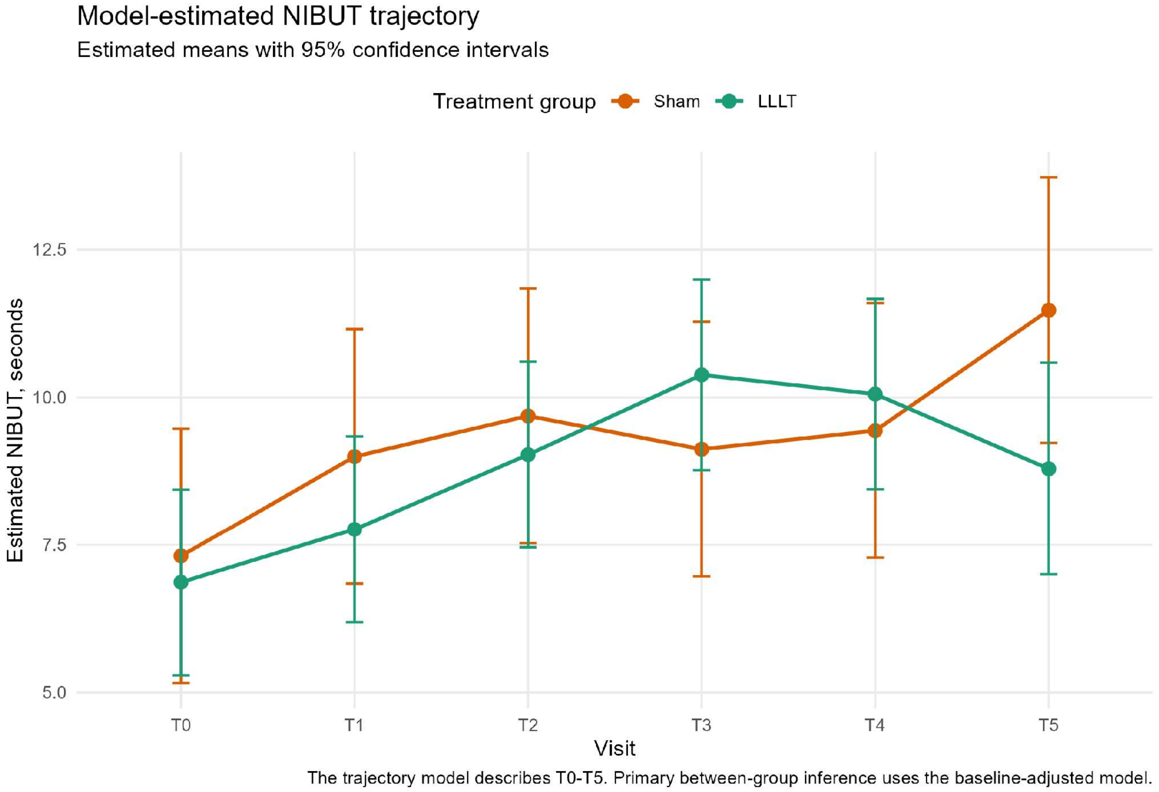

Supplementary Figure S2. Non-invasive break-up time (NIBUT) trajectory across time points, by treatment group.

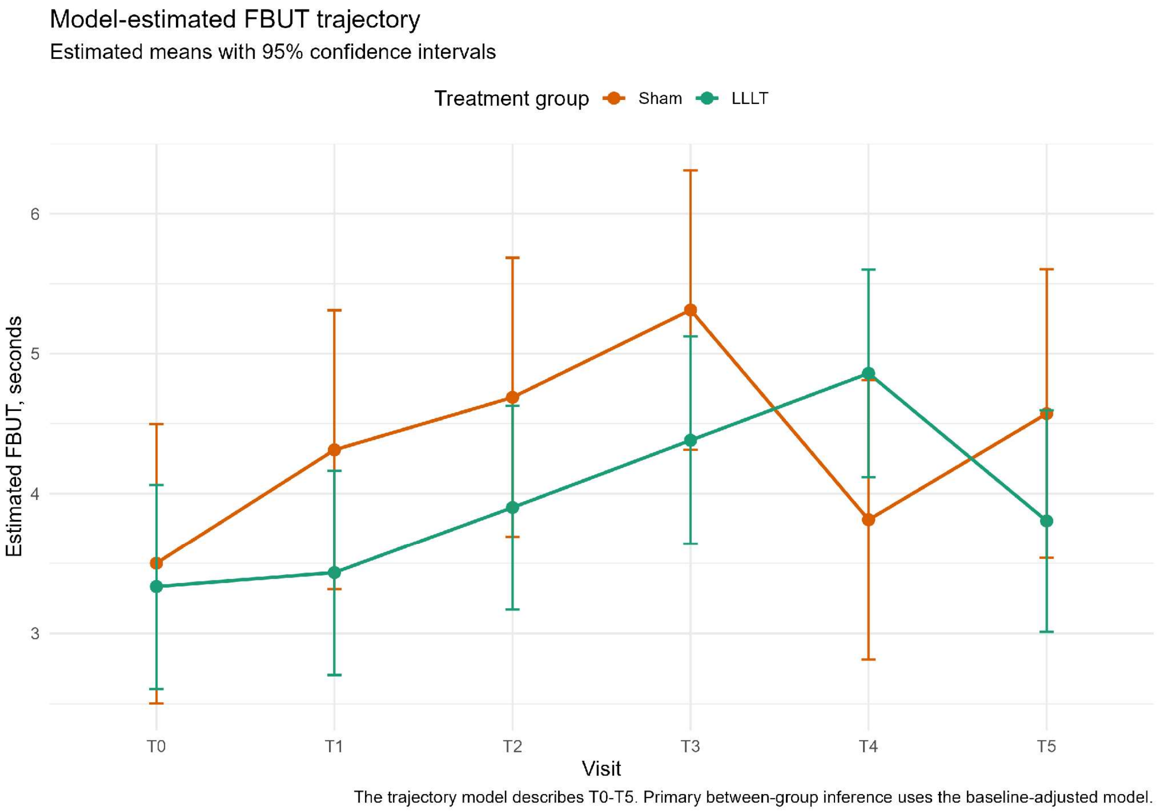

Supplementary Figure S3. Fluorescein break-up time (FBUT) trajectory across time points, by treatment group.

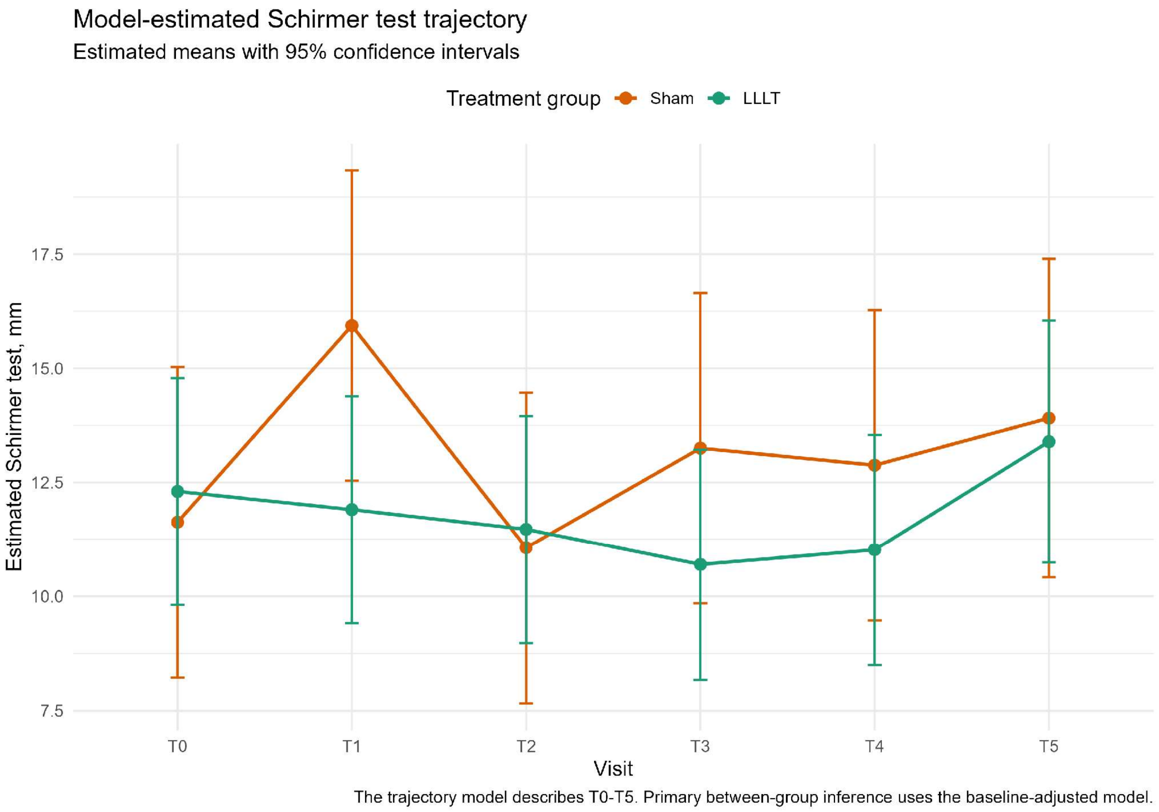

Supplementary Figure S4. Schirmer I test trajectory across time points, by treatment group.

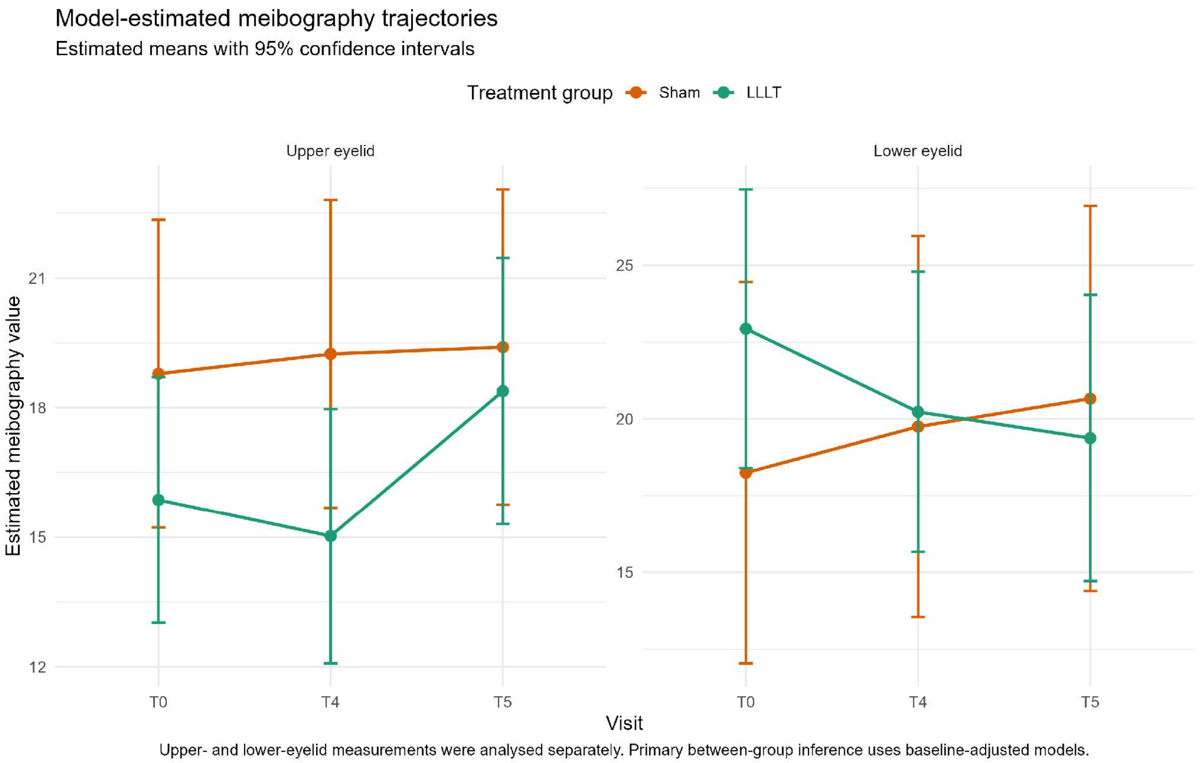

Supplementary Figure S5. Upper- and lower-eyelid meibography trajectories across time points, by treatment group.

Supplementary Table S1. Sensitivity analyses for the primary OSDI model (mean difference between groups, LLLT minus sham, with 95% confidence intervals).

Analysis	T4	T5
Mixed model, all available data	−11.97 (−16.44 to −7.50)	−16.86 (−21.51 to −12.21)
Mixed model, complete cases	−12.63 (−17.46 to −7.81)	−16.65 (−21.47 to −11.82)
ANCOVA, separate endpoint analyses	−11.76 (−16.90 to −6.63)	−16.65 (−21.81 to −11.49)
